# Multiple Proteases to Localize Oxidation Sites

**DOI:** 10.1371/journal.pone.0116606

**Published:** 2015-03-16

**Authors:** Liqing Gu, Renã A. S. Robinson

**Affiliations:** Department of Chemistry, University of Pittsburgh, Pittsburgh, PA, 15260, United States of America; Hokkaido University, JAPAN

## Abstract

Proteins present in cellular environments with high levels of reactive oxygen and nitrogen species and/or low levels of antioxidants are highly susceptible to oxidative post-translational modification (PTM). Irreversible oxidative PTMs can generate a complex distribution of modified protein molecules, recently termed as proteoforms. Using ubiquitin as a model system, we mapped oxidative modification sites using trypsin, Lys-C, and Glu-C peptides. Several M+16 Da proteoforms were detected as well as proteoforms that include other previously unidentified oxidative modifications. This work highlights the use of multiple protease digestions to give insights to the complexity of oxidative modifications possible in bottom-up analyses.

## Introduction

Reactive oxygen and nitrogen species in cellular environments can result in macromolecular damage [1]. Protein tertiary structure can be modified as a result of oxidative attack and lead to loss or gain of protein function [2]. Site-specific modifications can include the incorporation of oxygen atoms to amino acid side chains (e.g., methionine, phenylalanine, tyrosine, leucine, histidine), conversion to aldehydes, ketones, or carboxylic acids, addition of nitrogen containing groups (e.g., R-NO, R-NO_2_), formation of advanced glycation end products (e.g. imidazolinones, pyrralines), and introduction of lipid peroxidation products (e.g., 4-hydroxy-trans-nonenal, malondialdehyde) [3–5]. Key to understanding the events that affect protein function is the ability to characterize the distribution of oxidized proteoforms.

Techniques for the identification of proteoforms have been recently discussed [6–10]. Proteoforms can include molecules that arise due to the same post-translational modification (PTM) occurring at different amino acid residue positions in the protein. For example, a protein that incorporates a single oxygen atom during a free radical attack from hydrogen peroxide (H_2_O_2_) or superoxide anion, may exist in multiple locations. One population of the protein molecules can incorporate the oxygen at residue “A”, others incorporate at residue “B”, while the remaining molecules incorporate at both “A” and “B”. For the molecules with only a single oxygen addition, mass spectrometry (MS) measurements of intact protein would only detect a single M+16 Da species. Liquid chromatography (LC) or electrophoresis separations may be able to resolve the two proteoforms (i.e., A and B), however multiple dissociation methods such as collision activation dissociation (CAD) [11], infrared multiphoton dissociation [12], electron capture dissociation (ECD) [13], or electron transfer dissocation (ETD) [14] are necessary to localize the modification site.

Top-down and bottom-up protein analysis provides complementary information regarding protein sequence and PTMs [8,15–18], especially for identification of oxidation sites [19–21]. Top-down MS has been employed to characterize four oxidation sites in viral prolyl-r-hydroxylase [20] and for the identification of 250 isoforms of oxidized calmodulin [21]. Bottom-up proteomics is very useful for the verification of PTM types and sites although it can be challenging to identify the specific proteoform from which peptides originated. This is because shotgun analysis of all proteins extracted will lead to many similar peptides produced from various proteoforms. Because fragmentation of peptides is very accessible with CID and other dissociation methods, as compared to intact proteins, it is very practical to use bottom-up analyses to localize sites of oxidative modification. However, for complex biological samples the number of modification sites that can be characterized without extensive enrichment or separation strategies is generally low [22,23]. Therefore, it will be necessary to incorporate enrichment with our strategy of multiple proteases and iterative database searching to gain localize oxidative modification sites and obtain insight to the complexity of oxidized proteoforms present in complex mixtures.

Ubiquitin is a low molecular weight protein which has significant roles in protein turnover and degradation through its molecular chaperoning activity in the proteasome [24]. Ubiquitin is implicated in oxidative stress and disease [25,26]. The 76 amino acid sequence of this protein is highly conserved amongst eukaryotes, such as bovine and human [27]. Herein, we aimed to characterize the heterogeneity of proteoforms of ubiquitin using moderate oxidizing conditions [28] and bottom-up MS with multiple proteases. Chemical oxidizing conditions using Fenton chemistry [Fe(II)/H_2_O_2_] [28] rely on the metal serving as an electron donor to catalyze the formation of highly reactive hydroxyl radical (·OH) which can result in modification of amino acid side chains [5,29]. Previous studies have investigated oxidized forms of ubiquitin after exposure to peroxynitrite [30], electrochemical oxidation [31,32], and photochemical reactions [33] for the purpose of structural footprinting. The influence of N-terminal oxidation of Methionine (hereafter referred to as Met1-Ox) on protein structures and stabilities were examined by ion mobility spectrometry-mass spectrometry (IMS-MS)[34] and indicate that oxidation of Met1 can lead to destabilization of the native state and result in unfolded structures. Thus simple oxidized proteoforms can have a huge influence on protein structure.

Bottom-up LC-MS/MS of peptides generated from multiple proteases [35] allows multiple oxidation products of ubiquitin to be identified, including several proteoforms of the M+16 Da peak. Under Fe(II)/H_2_O_2_ conditions, numerous amino acid modifications are possible [36] and include side chain hydroxylation, carbonylation and backbone cleavage. The variety of these modifications requires multiple database searches be performed [37]. Sample integrity was confirmed by using high resolution ESI-MS on an Orbitrap Velos of intact oxidized protein mixtures.

## Experimental Methods

### In vitro Oxidation of Ubiquitin

Bovine ubiquitin was purchased from Sigma-Aldrich (St. Louis, MO). Protein (10 mg·mL^−1^) was dissolved in 10 mM sodium phosphate buffer solution (pH 7.4) and 10 mM H_2_O_2_ and 1 mM FeCl_2_ were added and allowed to react at 37°C for 2 hours. The reaction was quenched by flash freezing with liquid nitrogen. Protein sample was desalted on an HLB cartridge (Waters; Milford, MA) according to manufacturer’s instructions. Solvent was removed by centrifugal evaporation and dried protein stored at −80°C until further analysis.

### Top-down ESI-MS and MS^n^ Analysis

Intact oxidized ubiquitin (∼30 μM) was solubilized in 49:49:2 water:methanol:acetic acid. ESI-MS analysis was performed on a LTQ-Orbitrap Velos mass spectrometer (Thermo-Fisher Scientific, Waltham, MA) with direct infusion by a syringe pump. The following electrospray ionization parameters were used: spray voltage 4.25 kV; capillary temperature 200.00°C and flow rate 3 μL·min^−1^. Orbitrap detector settings included resolving power of 100 k, parent *m/z* scan range 600–2000, 3 μscans, and 30 and 100 scans for parent and fragmentation spectra, respectively. MS/MS data were recorded in the FT. MS/MS and MS^3^ settings used an isolation width of 1 *m/z* and normalized collision energy of 35%.

### Protein digestion

Purified oxidized ubiquitin (1 μg·μL^−1^) was solubilized in a denaturing buffer (0.2 M Tris, 8 M urea, 10 mM CaCl_2_, pH 8.0). Tris buffer (0.2M Tris, 10mM CaCl2, pH = 8.0) was added to dilute urea to 2M. The solution was separated into three equal volume aliquots and each incubated with TPCK-treated trypsin (Sigma), glutamic acid-C [(Glu-C); Princeton Separation, Inc, Adelphia, NJ] or lysine-C [(Lys-C); Princeton Separation, Inc] proteases at a 1:50 protein:enzyme mass ratio for 24 h at 37°C. Liquid nitrogen was used to quench digestions and samples were acidified by adding formic acid, desalted with HLB cartridges and the eluent dried by centrifugal evaporation.

### Nanoflow LC-MS/MS

Online desalting and reversed-phase chromatography was performed with a nanoLC system equipped with an autosampler (Eksigent; Dublin, CA). Mobile phases A and B for these analyses were 96.95:2.95:0.1 water:acetonitrile:formic acid and 99.9:0.1 acetonitrile:formic acid, respectively. Five μL of each peptide sample (1 μg·μL^−1^ in 0.1% formic acid) was loaded on to a trapping column [100 μm i.d. × 2 cm; 3 μm C_18_ 200 Å stationary phase material (Michrom Bioresource Inc.;Auburn, CA)] at 3 μL·min^−1^ in 3% mobile phase B for 3 min. After desalting, the sample was loaded onto a pulled-tip (using a CO_2_ laser) analytical column (75 μm i.d. × 13.2 cm), packed in-house with 3 μm C_18_ 100 Å stationary phase material (Michrom Bioresource Inc.). The following gradient was delivered at a flow rate of 300 nL·min^−1^: 0–5 min, 10% mobile phase B; 5–15 min, 10–30% B; 15–45 min, 30–45% B; 45–50 min, 45–60% B; 50–55 min, 60–80% B; 55–65 min, 80% B; 65–75 min, 10% B. The LC eluent was introduced into the ESI source with ∼1.5–2.0 kV. Data-dependent acquisition parameters were: parent Orbitrap MS resolving power 60 k; *m/z* scan range 300–1800; the top eight most intense ions were selected and activated using CID; isolation width 3 *m/z*; normalized collision energy 35%; dynamic exclusion was enabled with a repeat count of two for a duration of 60 sec; and, a minimum of 5000 ion counts for MS/MS. Samples were analyzed in triplicate.

### Data Analysis

Top-down spectra were viewed and analyzed by Xcalibur 2.1 software (Thermo). The Xtract program (Xcalibur) was used to deconvolute the spectra and calculate protein masses. Spectra were manually inspected and the *m/z* values matched to theoretical *b*- and *y*-type ions generated by ProteinProspector v5.9.4 [38]. For peptide data, .RAW files were analyzed with Proteome Discoverer 1.3 software (Thermo) and MS/MS spectra searched against a .fasta file containing the ubiquitin sequence (truncated from the N-terminal region of Uniprot ID P0CH28). Sequest search parameters included two maximum enzyme miscleavages; precursor mass tolerance of 10 ppm; fragment mass tolerance of 0.8 Da; dynamic modifications (see S1 Table) of mono oxidation to Lys, Arg, Pro, Thr, Met, His, Tyr, Ala, Asn, Asp, Glu, Gln, Ile, Leu, Phe, Ser and Val (Ox, +15.995 Da), dioxidation to Lys, Arg, Pro, His, Tyr, Asn, Asp, Phe and Met (DiOx, + 31.990 Da), carbonylation to Lys, Arg, Pro, Glu, Gln, Leu, Ser, Val and Ile (+13.979 Da), deamidation to Gln, Arg and Asn (0.984 Da), decarboxylation to Asp and Glu (-30.010 Da), oxidation of His to Asn (-23.0159 Da) or Asp (-22.032 Da) or aspartylurea (-10.032 Da) or ring open (4.979 Da), carbonylation of Arg to glutamic semialdehyde (GluSA, -43.053 Da), Lys to aminoadipic semialdehyde (AminoAdSA, -1.032 Da) or aminoadipic acid (14.963 Da), Pro to pyrrolidinone (-30.010 Da), and Thr to 2-amino-3-oxo-butanoic acid (Oxd’n, -2.016 Da). Only peptides with medium (p<0.05) and high confidence (p<0.01) as determined from a reverse decoy database search (which in Proteome Discoverer sets appropriate thresholds for XCorr values as a function of charge state) were used for initial filtering of the data [39–41]. For final inclusion of peptide hits and localization of modification sites, all MS/MS spectra were manually validated.

## Results and Discussion

### MS Analysis of Intact Oxidized Ubiquitin

**Fig 1:**
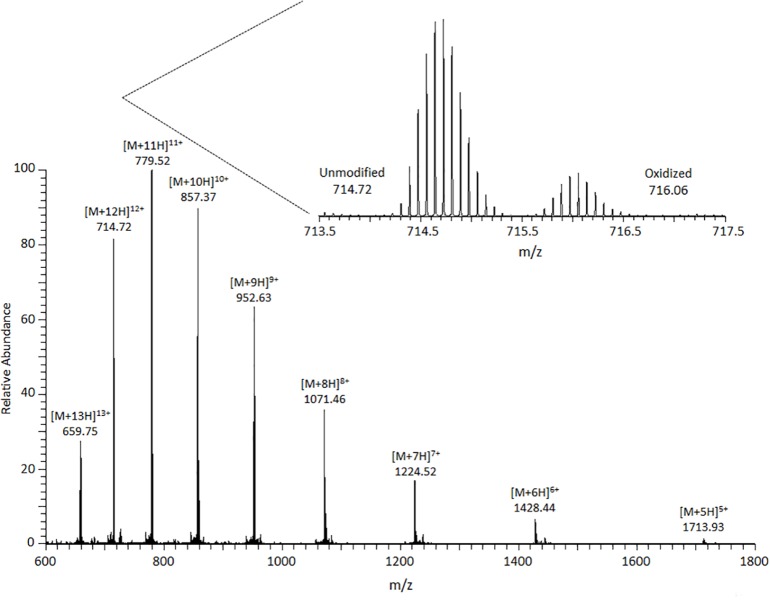
Precursor ion mass spectra of oxidized ubiquitin. In the inset is a zoom-in of the +12 charge state that shows unmodified and oxidized ubiquitin species. The observed mass shift between native and oxidized ubiquitin is indicated in the figure.

High-resolution ESI-MS analysis of a solution containing only untreated ubiquitin shows an M+16 Da peak that represents <2% of the total unmodified peak intensity in a deconvoluted spectrum (*data not shown*). Because the untreated sample has very limited sample handling, the M+16 Da species could be from the manufacturing and storage of the protein product, or from the electrospray ionization, (e.g. solution contact with metal needle or the electrolysis of water under high spray voltage. However, utilizing Fenton chemistry, several peaks belonging to oxidized ubiquitin are observed (Fig. 1). The most intense oxidized peak belongs to an M+16 Da species at each charge state measured (i.e., +5-+13). The inset of Fig. 1 shows a zoom-in of the +12 charge state, whereby two protein isotopic distributions are measured for unmodified and oxidized ubiquitin ions. Upon deconvolution of the spectrum it is noted that the M+16 Da species constitutes ~20% of the unmodified abundance. This is a factor of ten increase in M+16 Da ions in comparison to solutions containing only untreated ubiquitin. The masses of the deconvoluted native and M+16 Da peaks are 8564.601 and 8580.592 Da. These values are <5 ppm of the theoretically derived mass values and indicate a mass shift of 15.991 Da. Notably, this shift corresponds to the predominance of monooxygenated proteoforms in the Fe(II)/H_2_O_2_-ubiquitin mixture. The incorporation of an oxygen atom does not appear to influence the ionization efficiencies and hence observed signal intensity of ubiquitin in ESI [42], and is directly related to the relative abundance of each proteoform. Other proteoforms are observed in the spectrum: M-114 Da, M-57 Da, M-44 Da, M-16 Da, M+32 Da species, M+48 Da and M+96 Da. However, these peaks are low intensity and it’s possible many proteoforms are not observed in this direct infusion experiment.

### Characterizing Methionine Oxidation Proteoform

**Fig 2:**
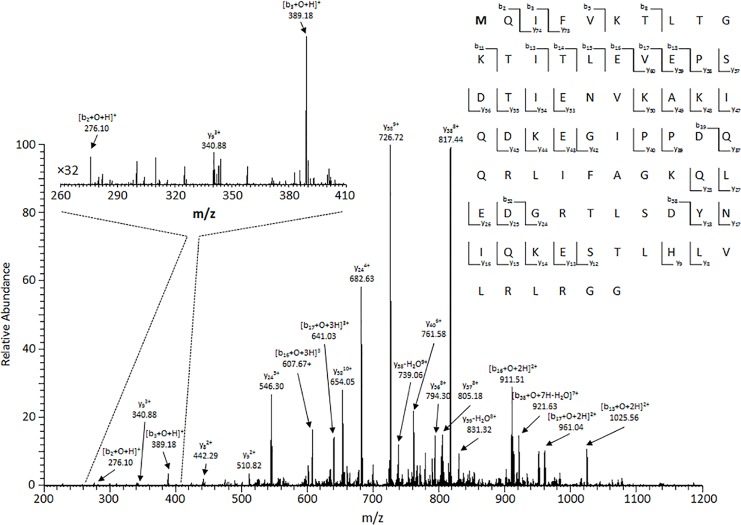
CID MS/MS spectra obtained upon isolation of +12 charge state oxidized ubiquitin species (*m/z* 716.06, isolation window 1 *m/z*). Zoom-in CID MS/MS spectra of the *m/z* range 260–410. To the right top is the sequence of ubiquitin with observed fragment ions across all *z* labeled.

Based on the sequence of ubiquitin, we anticipated methionine oxidation. The [M+O+12H]^12+^ protein peak was isolated and fragmented in the linear ion trap with CID. As shown in Fig. 2 many of the *b*- type fragment ion peaks are shifted in mass from expected fragments of unmodified ubiquitin ions by 16 Da. Moderate sequence coverage of the intact protein (Fig. 2) was obtained with CID and could be increased using dissociation methods such as ECD and ETD [43–46]. Inspection of the lower mass region (*m/z* 260–410) of the spectrum in Fig. 2 revealed the detection of [b_2_+O+H]^+^ and [b_3_+O+H]^+^ ions, indicating the oxygen atom addition on residue Met-1 or Gln-2. Based on the higher sensitivity of methionine to oxidation [47], it is very probable that the conversion of the single methionine residue to methionine sulfoxide occurred.

**Fig 3:**
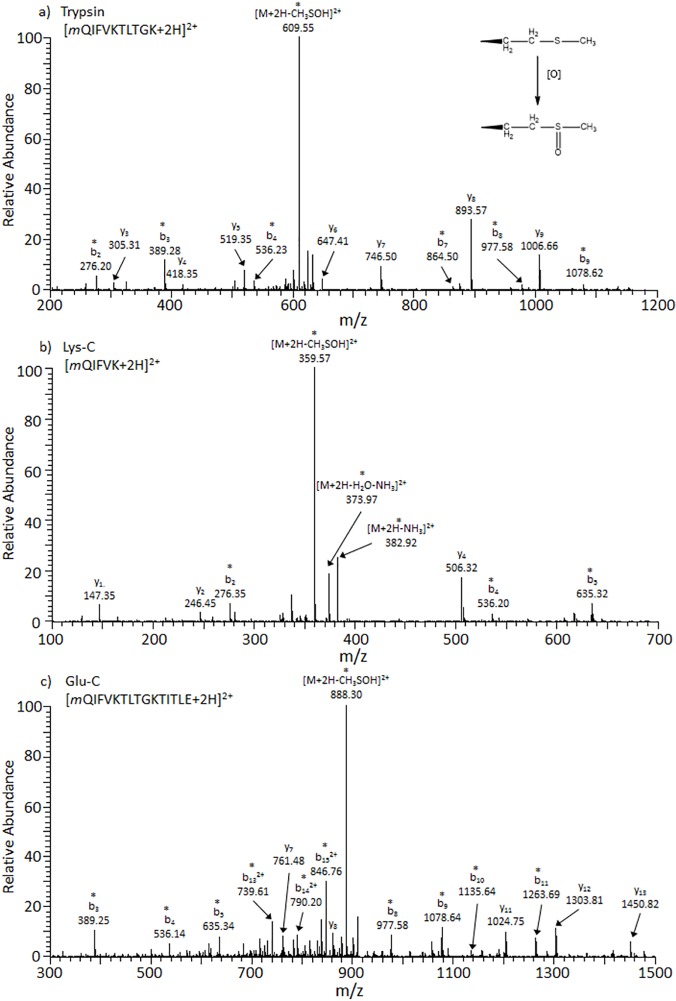
CID MS/MS spectra of (a) [mQIFVKTLTGK+2H]^2+^ with Met1-Ox as observed by trypsin proteolysis, t_r_ = 18.11 min, *m/z* = 641.36;(b) [mQIFVK+H]^2+^ with Met1-Ox as observed by Lys-C proteolysis, t_r_ = 21.27 min, *m/z* = 391.22 and (c) [mQIFVKTLTGKTITLE+2H]^2+^ with Met1-Ox as observed by Glu-C proteolysis, t_r_ = 26.58 min, *m/z* = 920.02. Note that lowercase letters represent the oxidation of methionine to methionine sulfoxide. Ions labeled with asterisks (*) contain modifications.

Multiple proteases allow for enhanced sequence coverage in proteomics as some cleavage sites are inaccessible with common enzymes [35] and oxidative modification may also hinder enzymatic cleavage for some residues (e.g., Lys and Arg) [48]. Peptide analysis of oxidized ubiquitin digests using trypsin, Lys-C, and Glu-C proteases is consistent with a M+16 Da methionine sulfoxide proteoform (see Table 1). Several oxidized methionine-containing peptides were identified in nanoLC-MS/MS analyses including doubly-charged mQIFVKTLTGK, mQIFVK, and mQIFVKTLTGKTITLE peptides derived from trypsin, Lys-C, and Glu-C proteases, respectively (Fig. 3). The most predominant peak in the MS/MS spectra of each of these peptides is the doubly-charged precursor ion with the loss of methyl sulfoxide [M+2H-CH_3_SOH]^2+^. This precursor ion is consistent with the presence of an oxidized methionine residue and has been observed by others [49,50]. For the spectra in Fig. 3, observed *b-*ions (including b_2_ fragments) are shifted by 16 Da which localizes the modification site to Met1. While not performed in these studies, methionine oxidation can also be tested through enzymatic action with peptidyl methionine sulfoxide reductase or by treatment with dithiothreitol [51,52].

**Table 1:** List of oxidative modifications identified from multiple proteases.

Sequence^*a*^	Modifications^*b*^	XCorr	Charge	m/z (Da)	MH+ (Da)	Δm (ppm)	t_r_ (min)	Protease
EGIPpDQQR^c^	P^38^-Oxidation	2.15	2	528.2591	1055.5109	-0.71	16.88	Trypsin
EGIPpDQQR^c^	P^38^-Carbonylation	2.39	2	527.2507	1053.4942	-1.73	15.80	Trypsin
EGIpPDQQR^c^	P^37^-Carbonylation	1.73	2	527.2507	1053.4941	-1.85	18.43	Trypsin
EGiPPDQQR^c^	I^36^-Carbonylation	2.18	2	527.2531	1053.4990	2.79	13.43	Trypsin
EGIPPDQQRLIfAGK	F^45^-Oxidation	3.03	2	842.9547	1684.9022	0.21	22.71	LysC
ESTLhLVLR^c^	H^68^-Oxidation	1.77	2	542.3115	1083.6158	0.04	24.09	Trypsin
ESTLHlVLR^c^	L^69^-Carbonylation	2.57	2	541.3021	1081.5969	-2.98	21.07	Trypsin
EStLHLVLR^c^	T^66^-Oxd'n	2.75	2	533.3046	1065.6019	-3.10	20.55	Trypsin
ESTLhLVLR^c^	H^68^-Asp	2.17	2	523.2972	1045.5872	-1.57	20.72	Trypsin
ESTLHlVlR^c^	L^69^-Oxidation, L^71^-Oxidation	2.14	2	550.3061	1099.6049	-5.22	22.16	Trypsin
ESTLhLVLR^c^	H^68^-Dioxidation	1.79	2	550.3055	1099.6037	-6.33	20.42	Trypsin
ESTLhLVLR^c^	H^68^-Histidine ring open (+5)	2.00	2	536.8010	1072.5947	-4.74	22.56	Trypsin
EstLHLVLRLRGG^c^	S^65^-Oxidation, T^66^-Oxd'n	2.32	3	488.9490	1464.8325	2.91	28.46	LysC
EVEPSDTIeNVKAKIQ^c^	E^24^-Decarboxylation	2.63	2	885.4650	1769.9227	-3.05	28.22	LysC
GkQLEDGR^c^	K^48^-Carbonylation	1.98	2	458.7275	916.4476	-0.78	14.51	Trypsin
IQDKEGIPpDQQR^c^	P^38^-Dioxidation	3.67	2	778.3890	1555.7707	-0.30	19.16	Trypsin
IQDKEGiPPDQQR^c^	I^36^-Carbonylation	2.55	3	513.2579	1537.7591	-0.98	14.30	Trypsin
LIfAGK	F^45^-Oxidation	1.79	1	664.4038	664.4038	1.31	22.16	Trypsin
LIfAGKQLEDGR^c^	F^45^-Oxidation	2.29	3	454.9167	1362.7355	-1.58	16.12	Trypsin
mQIfVK^c^	M^1^-Oxidation, F^4^-Oxidation	2.16	2	399.2154	797.4235	1.10	21.18	LysC
MQIfVK^c^	F^4^-Oxidation	1.97	2	391.2171	781.4269	-1.09	22.53	LysC
mQIFVK^c^	M^1^-Oxidation	1.77	1	781.4302	781.4302	3.23	22.04	GluC
mQIfVK^c^	M^1^-Oxidation, F^4^-Oxidation	2.13	2	399.2150	797.4228	0.18	21.01	Trypsin
MQIfVK^c^	F^4^-Oxidation	1.79	2	391.2177	781.4282	0.63	22.09	Trypsin
mQIFVK^c^	M^1^-Oxidation	1.72	1	781.4271	781.4271	-0.84	12.65	Trypsin
mQIFVK^c^	M^1^-Oxidation	1.96	2	391.2167	781.4261	-2.10	13.60	Trypsin
mQIFVK	M^1^-Oxidation	1.95	2	391.2177	781.4282	0.55	21.27	LysC
mQIFVK^c^	M^1^-Oxidation	1.76	1	781.4271	781.4271	-0.84	21.33	LysC
mQIFVKTL^c^	M^1^-Oxidation	2.84	2	498.2857	995.5641	4.63	25.08	GluC
MQIFVkTLTGK	K^6^-AminoAdSA	3.09	2	632.8536	1264.7000	2.35	26.95	LysC
mQIFVKTLTGK^c^	M^1^-Oxidation	3.05	3	427.9143	1281.7283	3.66	23.55	GluC
mQIFVKTLTGK	M^1^-Oxidation	3.52	2	641.3641	1281.7209	-2.12	18.11	Trypsin
mQIFVKTLTGK^c^	M^1^-Oxidation	2.80	3	427.9117	1281.7207	-2.27	18.12	Trypsin
mQIFVKTLTGKTITLE	M^1^-Oxidation	4.79	2	920.0217	1839.0362	3.53	26.58	GluC
mQIFVKTLTGKTITLEVEPSDTIENVKAK^c^	M^1^-Oxidation	5.18	4	813.1945	3249.7560	-2.84	22.06	Trypsin
NVKAKIQDkeG^c^	K^33^-Oxidation; E^34^-Decarboxylation	3.10	2	608.3413	1215.6752	4.92	18.97	GluC
NVKAKIQDKEGIPp^c^	P^38^-Dioxidation	2.54	3	523.6282	1568.8702	3.71	20.44	GluC
QLEDGRTLSDyNIQK	Y^59^-Oxidation	3.68	2	898.4461	1795.8849	1.55	22.14	LysC
QLEDGrTLSDYNIQK^c^	R^54^-GluSA	2.34	2	868.9171	1736.8270	-3.94	19.35	Trypsin
tITLEVEPSDTIENVK^c^	T^12^-Oxd'n	3.14	2	893.4609	1785.9145	1.54	25.96	LysC
TITlEVEPSDTIENVK^c^	L^15^-Oxidation	3.44	2	902.4661	1803.9249	1.42	24.32	Trypsin
TITLEVEPsDTIENVK^c^	S^20^-Carbonylation	2.83	2	901.4577	1801.9081	0.83	24.64	Trypsin
TITLEVePSDTIENVK^c^	E^18^-Decarboxylation	2.03	2	879.4589	1757.9105	-3.62	22.07	Trypsin
TLSDYNIQk^c^	K^63^-Oxidation	2.56	2	549.2772	1097.5472	-0.17	21.30	Trypsin
TLSDYNIqK^c^	Q^62^-Deamidation	2.17	2	541.7697	1082.5320	-4.09	14.47	Trypsin
TLSDyNIQK	Y^59^-Oxidation	2.61	2	549.2759	1097.5446	-2.51	14.33	Trypsin
tLSDYNIQK^c^	T^55^-Oxd'n	2.13	1	1079.5325	1079.5325	-4.00	19.29	Trypsin
tLSDYNIQK	T^55^-Oxd'n	1.74	2	540.2690	1079.5307	-5.65	20.28	Trypsin

^*a*^Lowercase letters represent the amino acid residues that have oxidative modifications.

^*b*^Positions of modified residues in the entire ubiquitin sequence are shown and are abbreviated as follows: Oxidation indicates an oxygen addition to the amino acid residue, Dioxidation indicated two oxygens addition to the amino acid residue, carbonylation indicates formaton of carbonyl group with a mass increase of 14 Da, GluSA indicates the carbonylation of arginine to glutamic semialdehyde, AminoAdSA indicates the carbonlyation of lysine to aminoadipic semialdehyde, Oxd'n indicates the carbonlyation of threonine to 2-amino-3-oxo-butanoic acid, Asp indicates the oxidation of histidine to aspartic acid, Deamidation indicates the conversion of -NH_2_ to -OH and Decarbonylation indicates the loss of carboxyl group.

^*c*^MS/MS spectra of each peptide is provided in S1 Fig.

### Mapping Other M+16 Da Proteoforms

**Fig 4:**
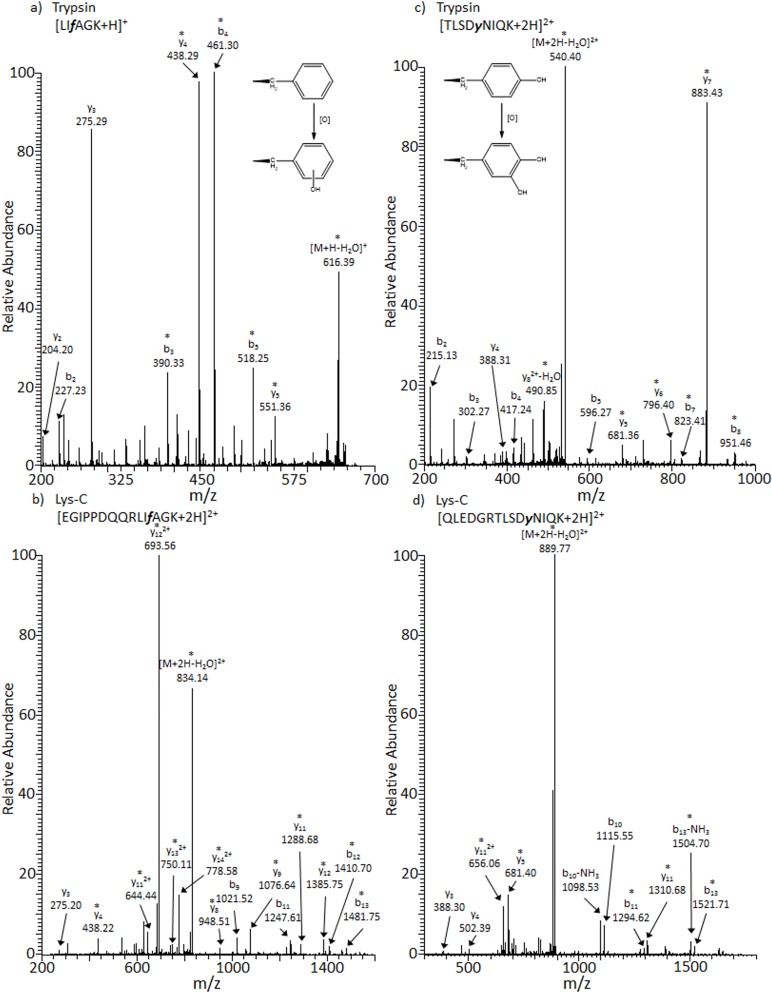
CID MS/MS spectra of (a) [LIfAGK+H]^+^ with Phe45-Ox as observed by trypsin proteolysis, t_r_ = 22.16 min, *m/z* = 664.40; (b) [EGIPPDQQRLIfAGK+2H]^2+^ with Phe45-Ox as observed by Lys-C proteolysis, t_r_ = 22.71, *m/z* = 842.95; (c) [TLSDyNIQK+2H]^2+^ with Tyr59-Ox as observed by trypsin proteolysis, t_r_ = 14.33 min, *m/z* = 549.28 and (d) [QLEDGRTLSDyNIQK+2H]^2+^ with Tyr59-Ox as observed by Lys-C proteolysis, t_r_ = 22.14 min, *m/z* = 898.45. Note that lowercase letters represent the oxidation of phenylalanine and tyrosine. Ions labeled with asterisks (*) contain modifications.

Fig. 4A and 4B show MS/MS spectra of the tryptic peptide [LIfAGK+H]^+^ and the Lys-C peptide [EGIPPDQQRLIfAGK+2H]^2+^, respectively. Each of these spectra contains fragment ions that localize an oxidative modification to a phenylalanine residue (i.e., Phe45). Phenylalanine contains an aromatic ring which upon oxidation can be modified by hydroxyl radicals at the *para-*, *ortho-*, or *meta-* positions as shown in Fig. 4A [53,54]. Similarly, MS/MS spectra of the tryptic peptide [TLSDyNIQK+2H]^2+^ and Lys-C peptide [QLEDGRTLSDyNIQK+2H]^2+^ tentatively assign oxidation of the Tyr59 residue (Fig. 4C and 4D, respectively).

Overall we observe several peptides which contain a monoxygenated residue (Table 1). Positions of these modifications are: Met1, Phe4, Leu15, Lys33, Phe45, Ser55, Tyr59, Lys63, His68, Leu69, and Leu71. It is possible that each of these peptides arise from different molecules of intact M+16 Da proteoforms. However, it is also likely that they arise from lower intensity M+32 Da or other oxidized proteoforms. Ambiguous identifications include Pro37 which is shifted by 16 Da and could correspond to oxygen incorporation or a carbonyl shift to glutamic semialdehyde.

### Identification of Other Oxidative Proteoforms

**Fig 5:**
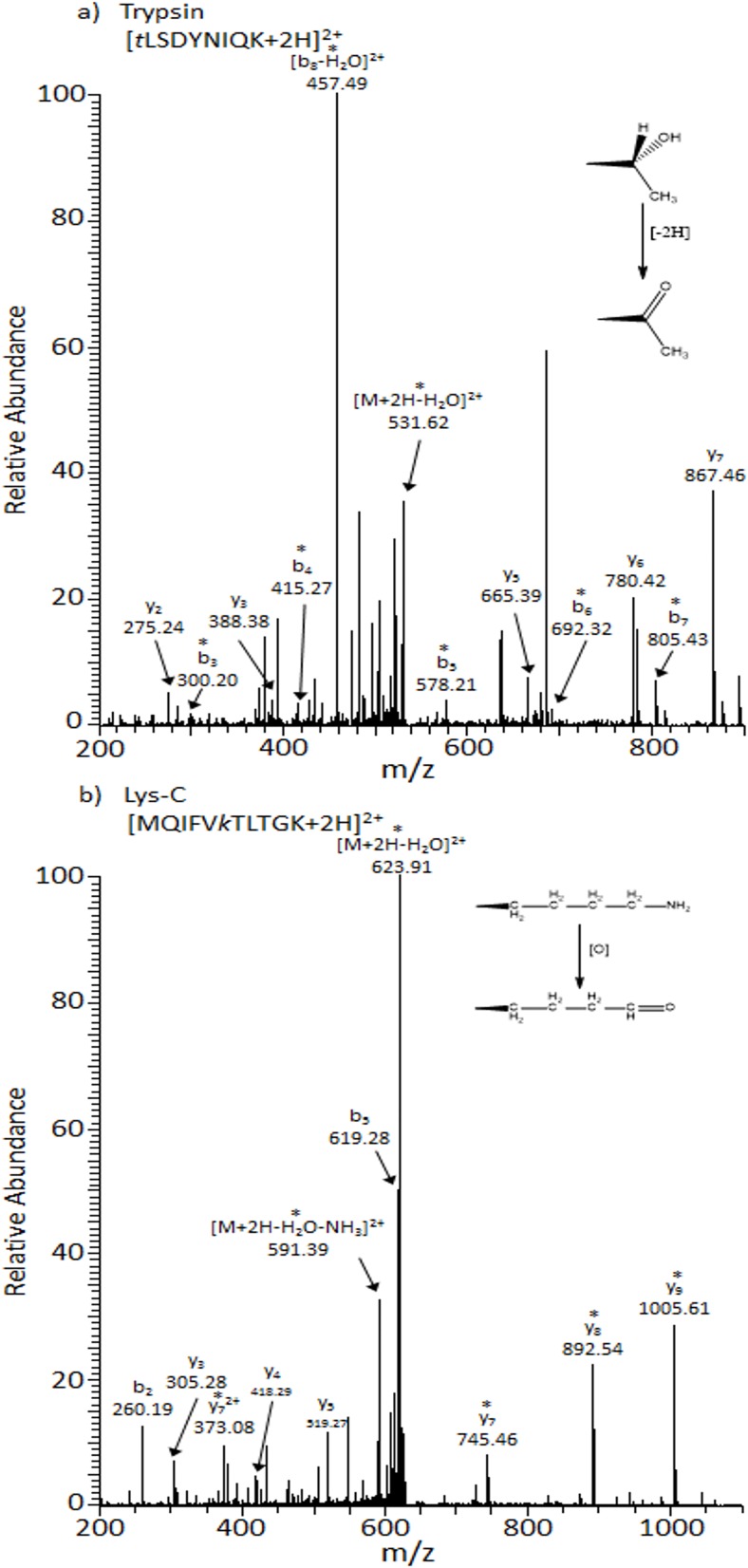
CID MS/MS spectra of (a) [tLSDYNIQK+2H]^2+^ with Thr55-Oxd’n as observed by trypsin proteolysis, t_r_ = 20.28min, *m/z* = 540.27 and (b) [MQIFVkTLTGK+2H]^2+^ with Lys6 – AminoAdSA as observed by Lys-C proteolysis, t_r_ = 26.95, *m/z* = 632.85. Note that lowercase letters represent the carbonylation of threonine to 2-amino-3-oxo-butanoic acid and lysine to aminoadipic semialdehyde. Ions labeled with asterisks (*) contain modifications.

The most abundant oxidized species that exists in these data arise from the incorporation of oxygen to amino acid side chains however other proteoforms are present. Fig. 5A and 5B are example MS/MS spectra from trypsin and Lys-C peptides [tLSDYNIQK+2H]^2+^ and [MQIFVkTLTGK+2H]^2+^, respectively. Fragment ions are present (Fig. 5A) which locate an oxidation site to threonine55. Threonine oxidation results in carbonylation to 2-amino-3-oxo-butanoic acid represented by a mass loss of −2.016 Da. Fig. 5B provides MS/MS fragments which identify Lys6 oxidation represented by a mass loss of 1.032 Da. It is noted that this N-terminal peptide contains an unmodified methionine residue and thus originates from other oxidized proteoforms. Other modifications observed with multiple proteases are provided in Table 1 (and S1 Fig.). It is possible that oxidative modifications present can influence MS/MS fragmentation patterns and this is dependent on particular amino acid and modification type. MS/MS spectra were manually inspected to eliminate ambiguous and low confidence assignments.

Extensive database searches were performed in order to search for many potential oxidative modifications that may occur using metal-catalyzed oxidation. In addition to searching for hydroxylation and carbonylation of Lys, Arg, Thr and Pro residues, our searches also included dioxidation, carbonylation on other amino acids, deamidation, decarboxylation, as well as different oxidative products of His (S1 Table). Examples of new modifications, e.g. ring opening of His68, carbonylation of Ser20, Ile36, Leu69, were successfully identified through these additional database searches (Table 1).

Fe(II)/H_2_O_2_ oxidation of ubiquitin leads to multiple M+16 Da and other proteoforms. The most abundant M+16 Da species contains Met1-Ox and is consistent with high oxidation reactivity [55]. M+16 Da and M+32 Da proteoforms were distinguishable using top-down MS^n^ however, CID MS/MS – MS^n^ only provided a limited amount of information about modification sites. Other dissociation methods such as ECD and ETD may provide more extensive sequence coverage and directly localize oxidative modification sites. On the other hand, bottom-up data only provide information about peptides that are observed in solution and do not completely reflect the specific oxidized proteoforms from which they originate. For example, the tryptic peptide mQIFVK could have arisen from an intact M+16 Da ubiquitin molecule or from other oxidized proteoforms. The observation of the tryptic peptide mQIfVK with two oxidized residues and the variety of modified residues listed in Table 1, clearly indicates that many oxidized proteoforms are present.

Thus use of multiple proteases greatly improved our ability to localize oxidative modification sites of oxidized ubiquitin. Specifically, six out of 24 modified sites identified by trypsin are validated by Lys-C and Glu-C experiments (e.g. Met1 is identified by all three proteases). Modifications to residues such as Lys6, Thr12, Glu24 and Ser65 were only identified with Lys-C and Lys33, Glu34 were only identified by Glu-C (See Table 1). We note that incorporation of an enrichment or tagging step may improve the detection of other oxidized proteoforms [21–23]. The combination of multiple proteases with iterative database searching was key to identification of so many oxidized sites and proteoforms of ubiquitin. Due to limitations with the number of modifications that can be simultaneously searched with SEQUEST (in Proteome Discoverer), we performed iterative database searches by classifying the modifications into seven groups (methionine oxidation, non-methionine oxidation, carbonylation (+14 Da), deamidation, decarboxylation, histidine oxidation and special carbonylation) [37]. This resulted in each RAW file being searched 22 times. For simple protein mixtures in which one seeks to gain knowledge about the complexity of oxidized proteoforms we present this strategy (combination of top-down mapping, multiple proteases, and iterative database searching) as an alternative to targeted chemistries or enrichment steps.)

## Conclusions

This work reports on the detection of oxidized species of Fe(II)/H_2_O_2_ oxidized ubiquitin molecules using multiple proteases and iterative database searching of oxidative modifications. Multiple proteases allowed numerous modification sites to be identified and increased confidence in each oxidative site. Multiple proteases also allowed inaccessible sites by trypsin digestion to be available with other proteases. Iterative database searching allowed different types of oxidative modifications to be identified, however this also required manual validation of MS/MS spectra and extended computing times. Under the mM concentrations of oxidizing reagent used, oxidative modifications to ubiquitin included protein carbonylation. The ability to identify distributions of proteoforms for simple systems, such as ubiquitin, using multiple proteases in shotgun proteomics can be extended to larger and more complex protein samples. Complex protein samples will benefit from additional enrichment steps. The utility of multiple proteases combined with iterative database searching of oxidative modifications can be extended to other types of PTMs such as glycosylation, cysteine oxidations, and amidation and has promising applications in pharmaceutical industries for the analysis of intact antibodies.

## Supporting Information

S1 Fig MS/MS spectra of oxidatively-modified peptides identified in Table 1. (PDF)

S1 Table List of the oxidative modifications included in the database search. (XLSX)
