# Supplementary material for: Multiple Proteases to Localize Oxidation Sites
Source: PLoS One. 2015 Mar 16;10(3):e0116606. doi: 10.1371/journal.pone.0116606 (PMC4361631; doi:10.1371/journal.pone.0116606)

Sequence	Modifications	XCorr	Charge	m/z (Da)	MH+ (Da)	Δm (ppm)	t_r (min)	Enzyme
EGIPpDQQR	P^{38}-Oxidation	2.15	2	528.2591	1055.5109	-0.71	16.88	Trypsin

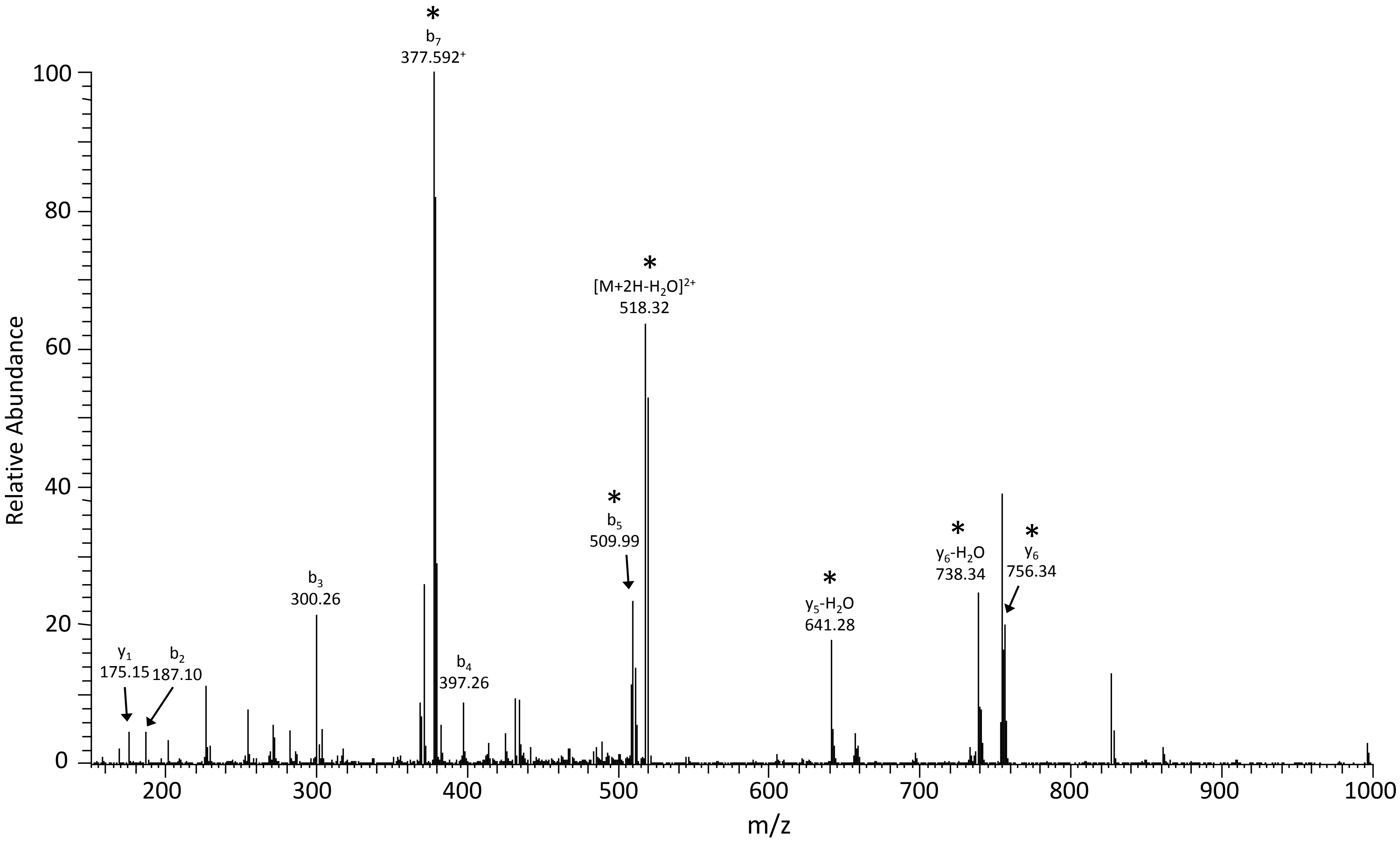

Sequence	Modifications	XCorr	Charge	m/z (Da)	MH+ (Da)	Δm (ppm)	t_r (min)	Enzyme
EGIPpDQQR	P^{38}-Carbonylation	2.39	2	527.2507	1053.4942	-1.73	15.80	Trypsin

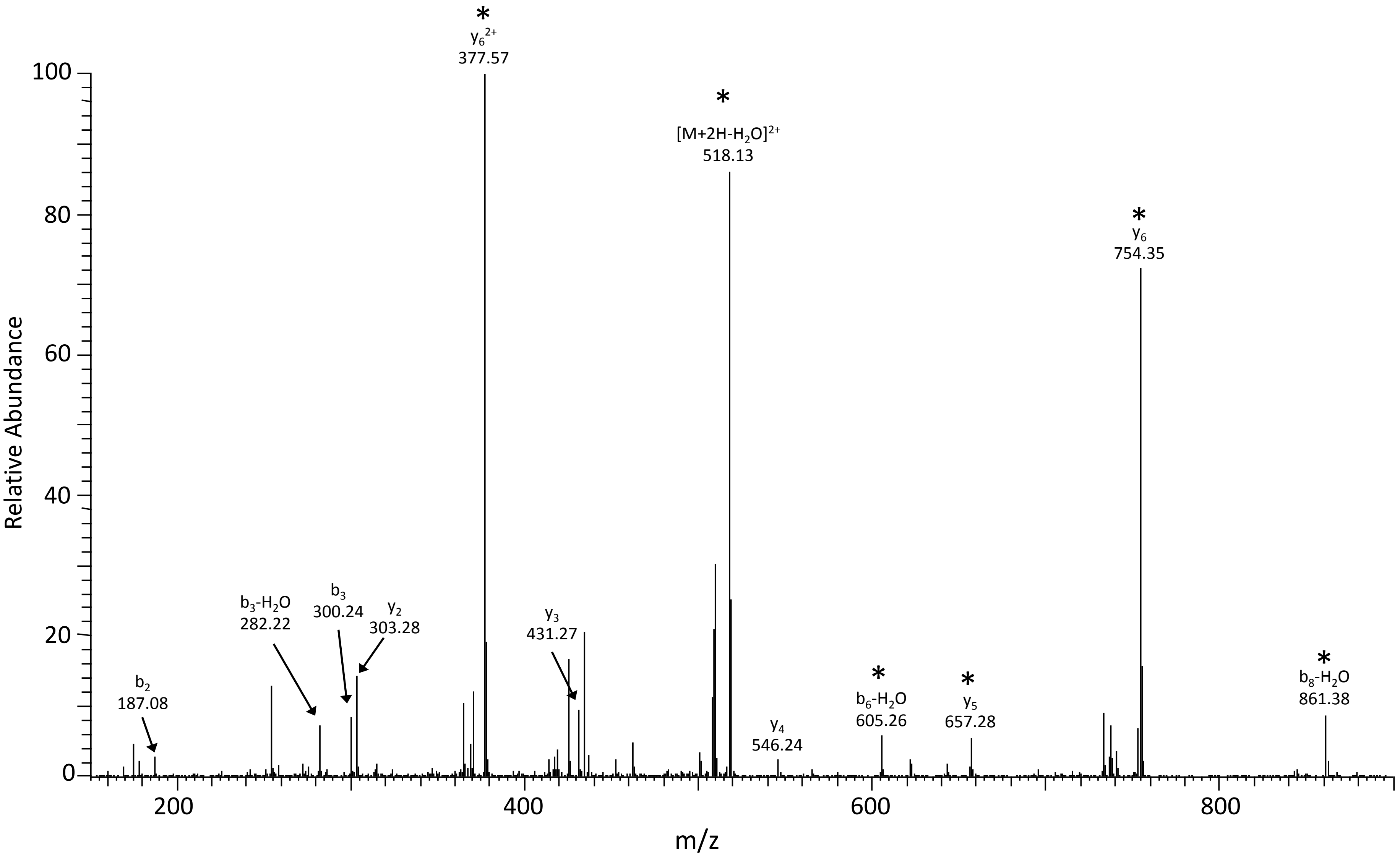

Sequence	Modifications	XCorr	Charge	m/z (Da)	MH+ (Da)	Δm (ppm)	t_r (min)	Enzyme
EGIpPDQQR	P^{37}-Carbonylation	1.73	2	527.2507	1053.4941	-1.85	18.43	Trypsin

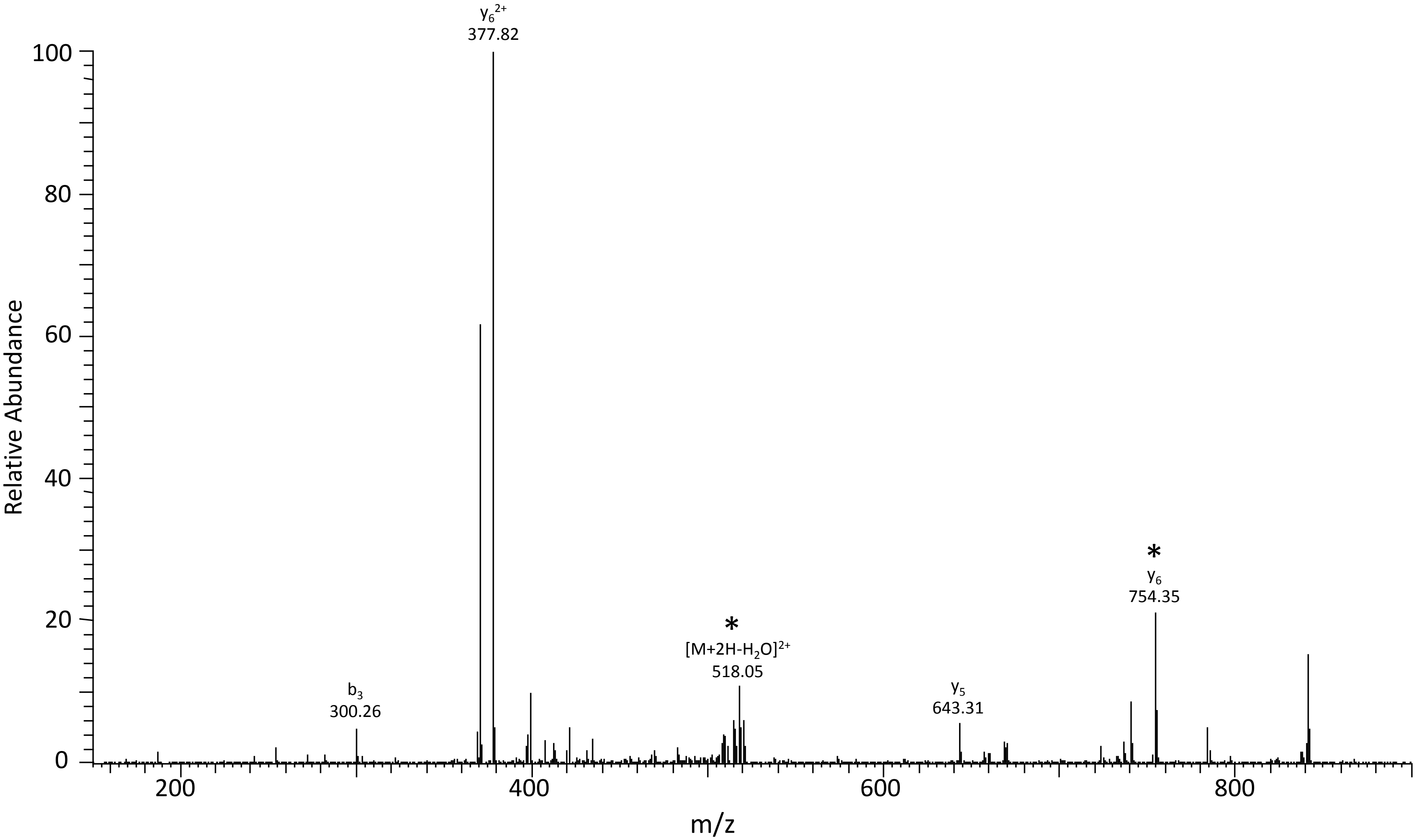

Sequence	Modifications	XCorr	Charge	m/z (Da)	MH+ (Da)	Δm (ppm)	t_r (min)	Enzyme
EGiPPDQQR	I^{36}-Carbonylation	2.18	2	527.2531	1053.4990	2.79	13.43	Trypsin

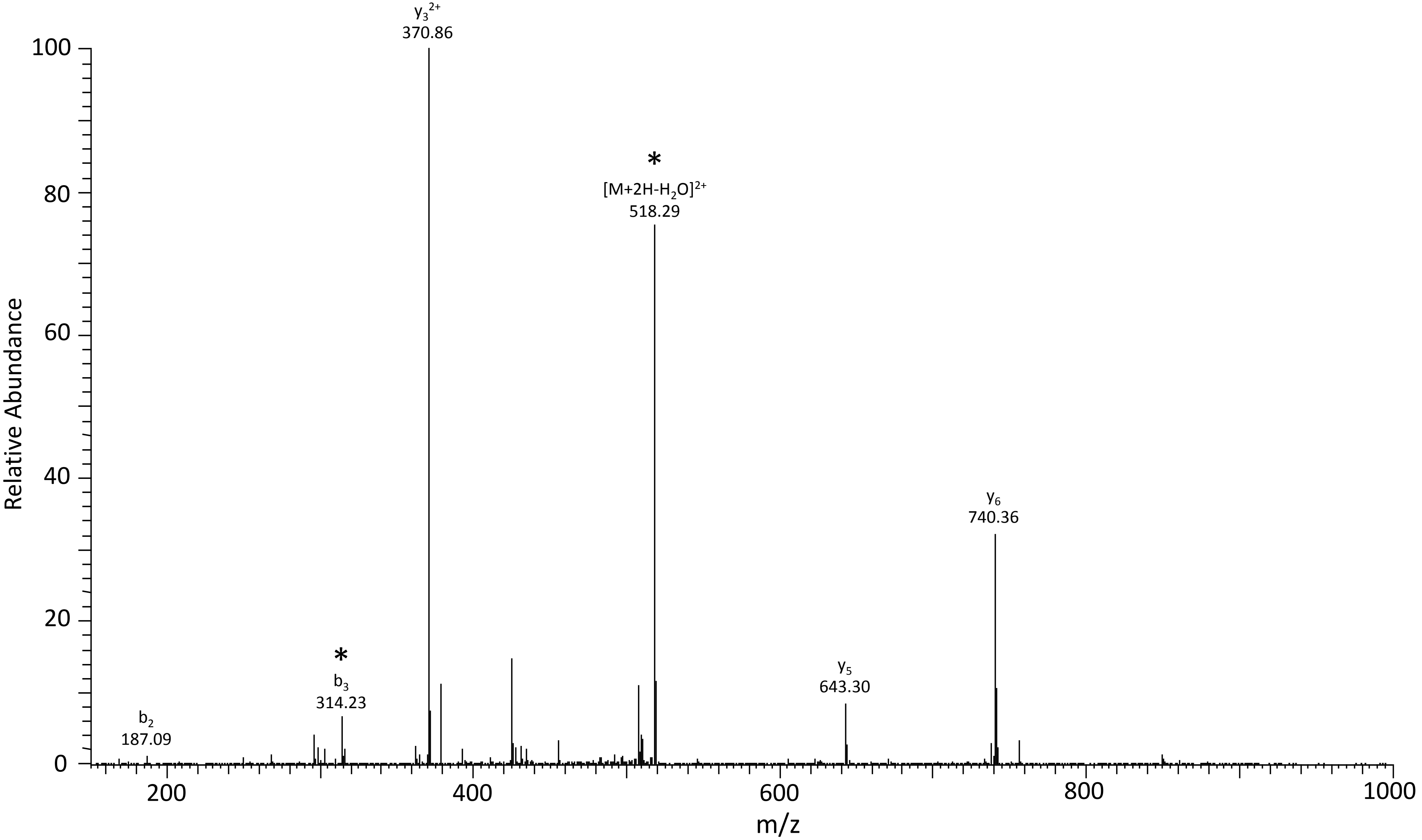

Sequence	Modifications	XCorr	Charge	m/z (Da)	MH+ (Da)	Δm (ppm)	t_r (min)	Enzyme
ESTLhLVLR	H^{68}-Oxidation	1.77	2	542.3115	1083.6158	0.04	24.09	Trypsin

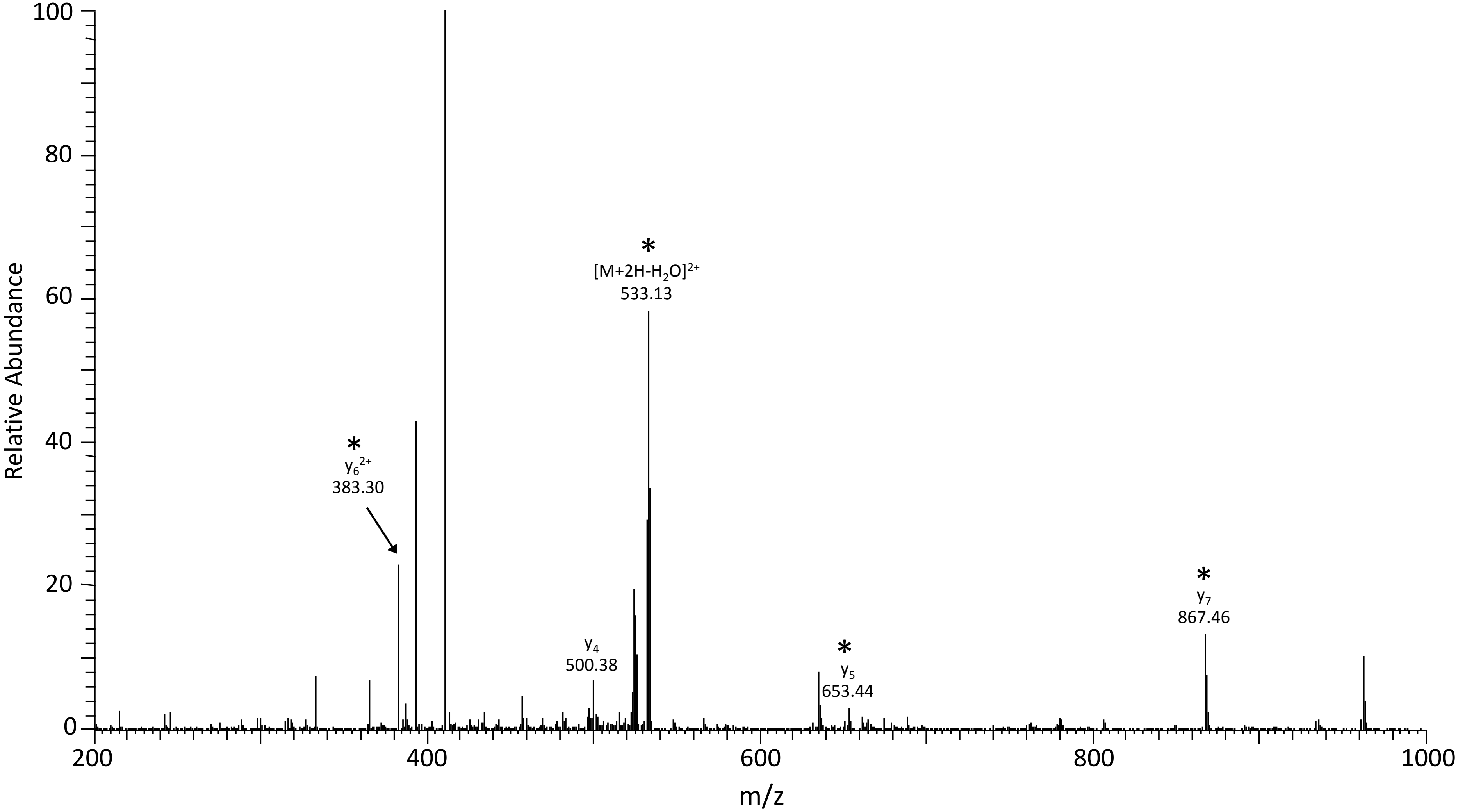

Sequence	Modifications	XCorr	Charge	m/z (Da)	MH+ (Da)	Δm (ppm)	t_r (min)	Enzyme
ESTLHIVLR	L^{69}-Carbonylation	2.57	2	541.3021	1081.5969	-2.98	21.07	Trypsin

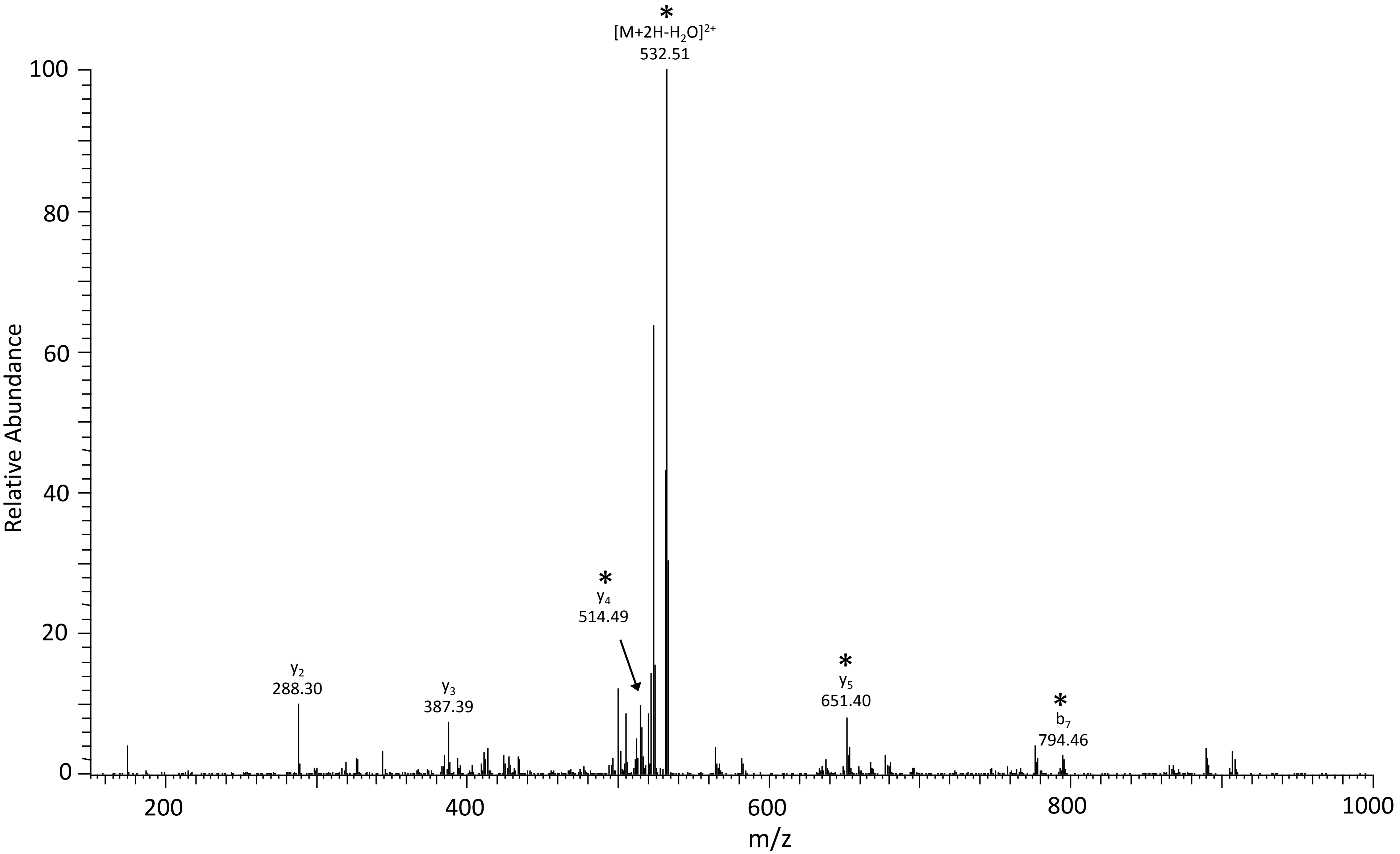

Sequence	Modifications	XCorr	Charge	m/z (Da)	MH+ (Da)	Δm (ppm)	t_r (min)	Enzyme
EStLHLVLR	T^{66}-Oxd'n	2.75	2	533.3046	1065.6019	-3.10	20.55	Trypsin

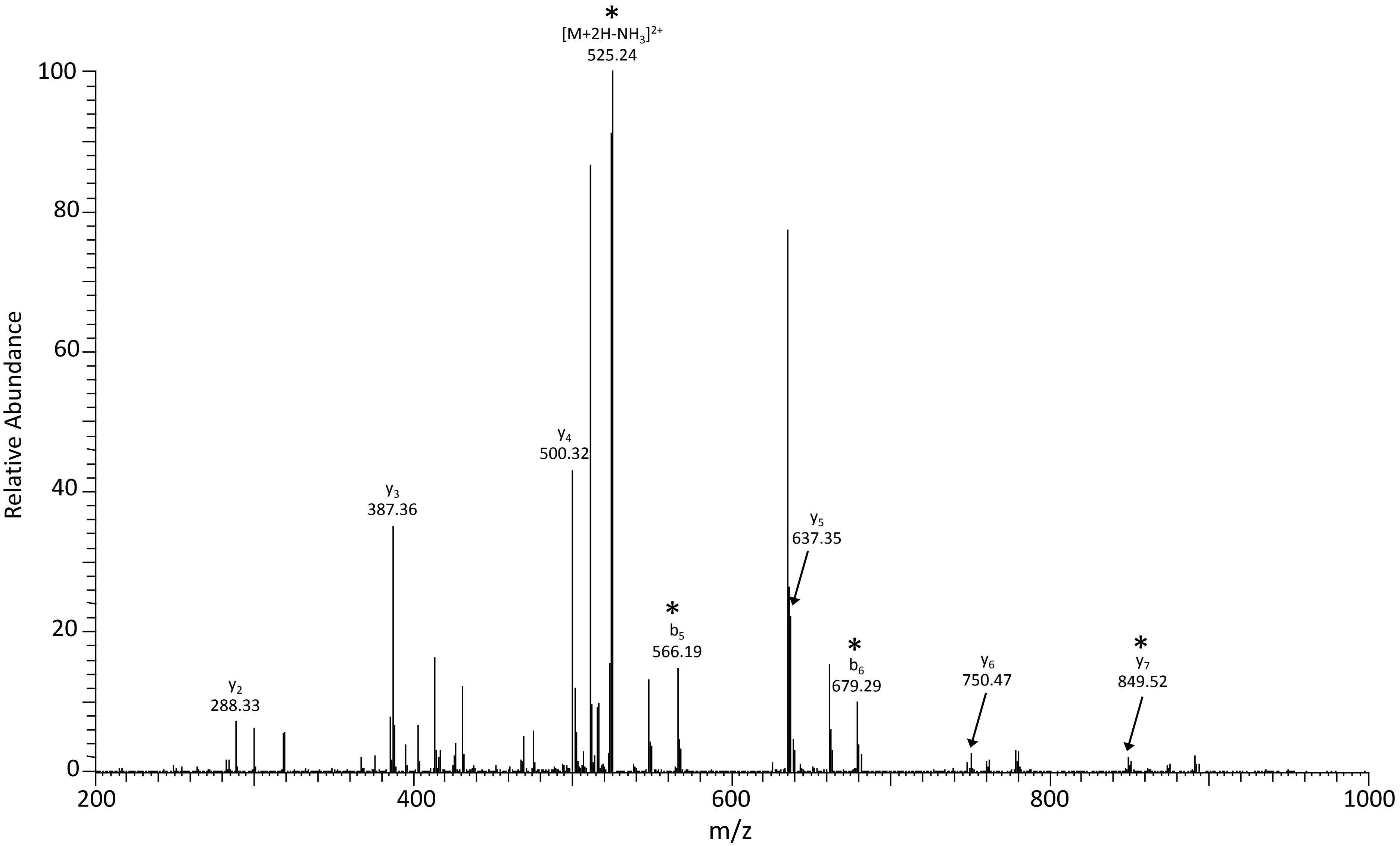

Sequence	Modifications	XCorr	Charge	m/z (Da)	MH+ (Da)	Δm (ppm)	t_r (min)	Enzyme
ESTLhLVLR	H^{68}-Asp	2.17	2	523.2972	1045.5872	-1.57	20.72	Trypsin

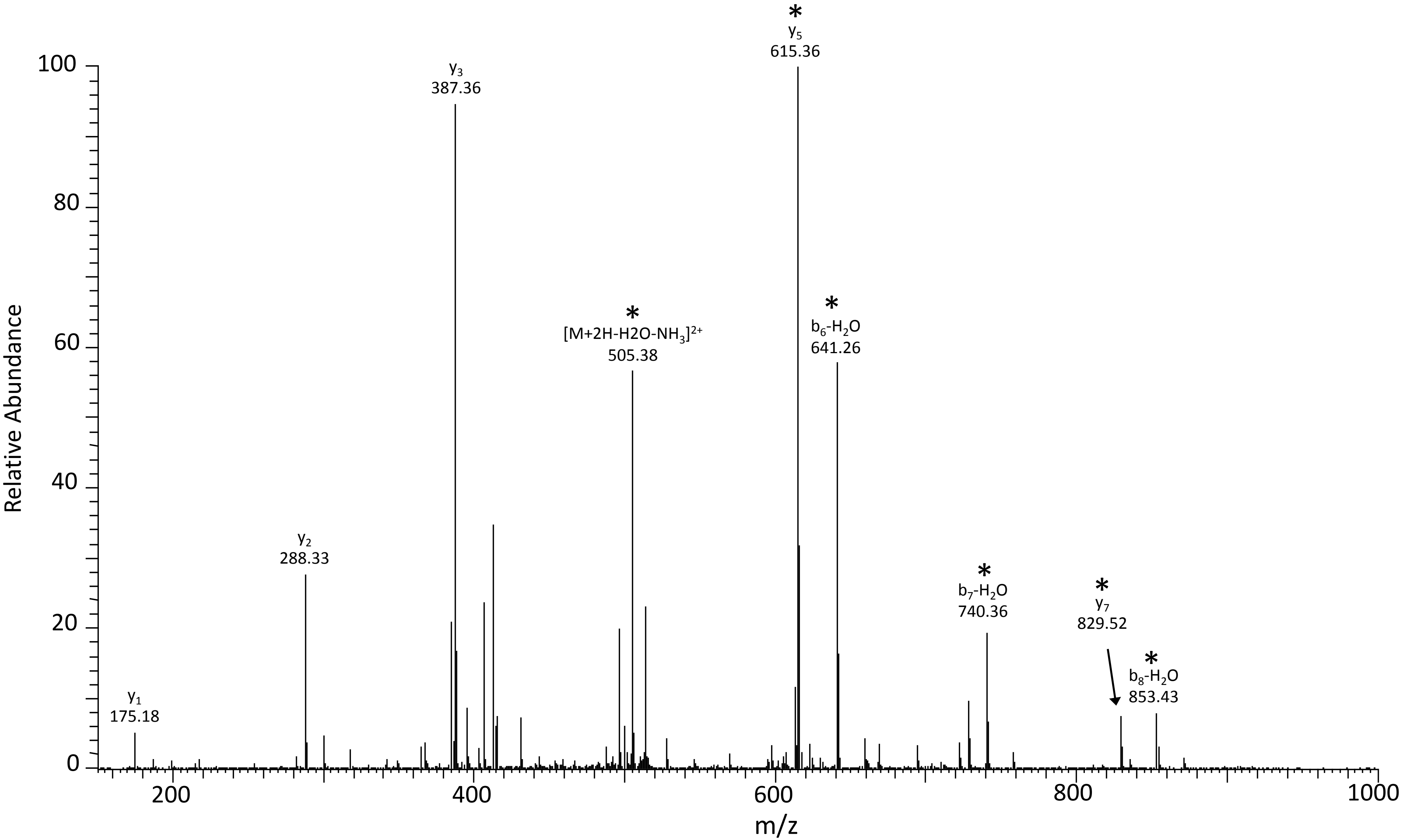

Sequence	Modifications	XCorr	Charge	m/z (Da)	MH+ (Da)	Δm (ppm)	t_r (min)	Enzyme
ESTLHlVlR	L^{69}-Oxidation, L^{71}-Oxidation	2.14	2	550.3061	1099.6049	-5.22	22.16	Trypsin

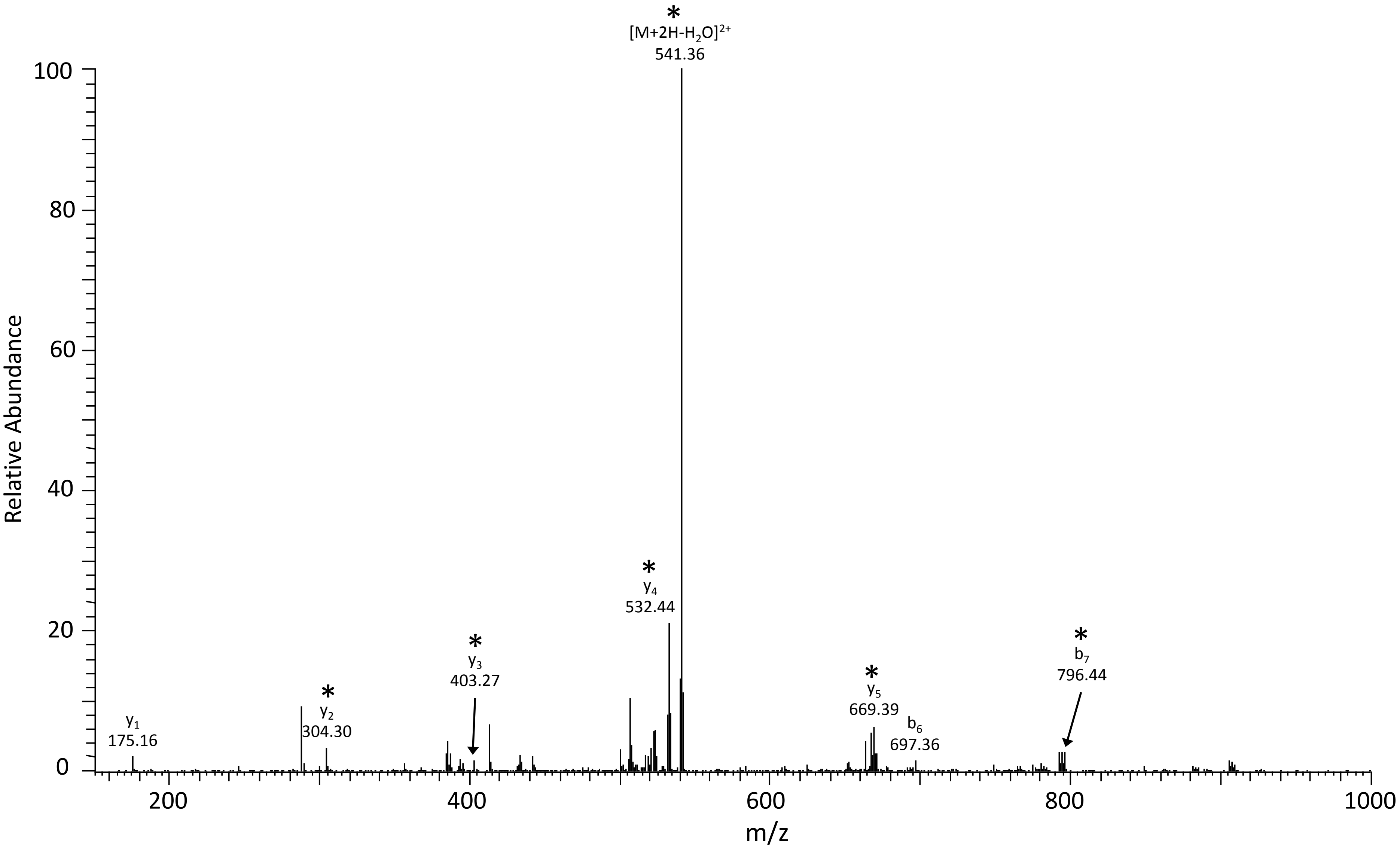

Sequence	Modifications	XCorr	Charge	m/z (Da)	MH+ (Da)	Δm (ppm)	t_r (min)	Enzyme
ESTLhLVLR	H^{68}-Dioxidation	1.79	2	550.3055	1099.6037	-6.33	20.42	Trypsin

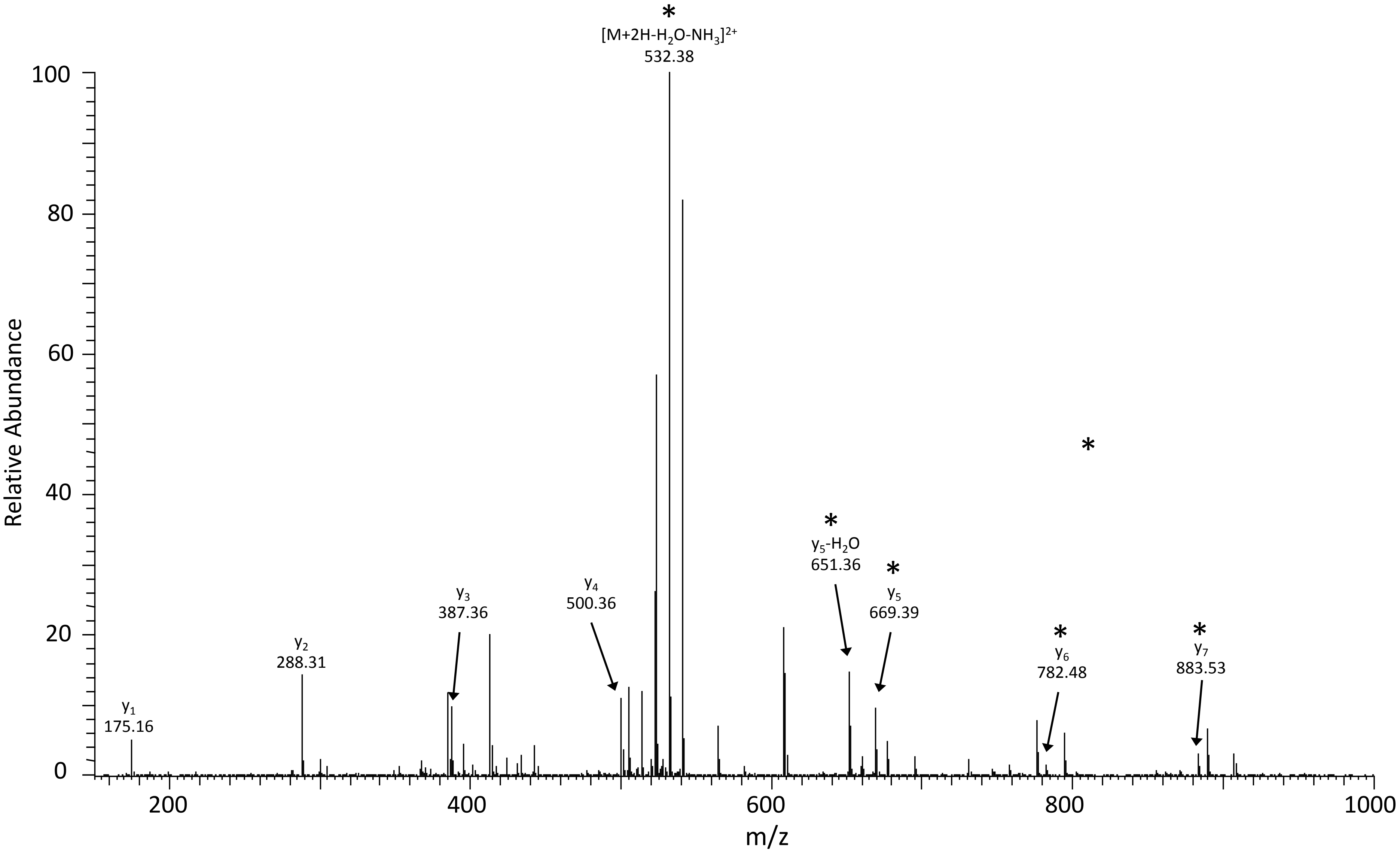

Sequence	Modifications	XCorr	Charge	m/z (Da)	MH+ (Da)	Δm (ppm)	t_r (min)	Enzyme
ESTLhLVLR	H^{68}-Histidine ring open (+5)	2.00	2	536.8010	1072.5947	-4.74	22.56	Trypsin

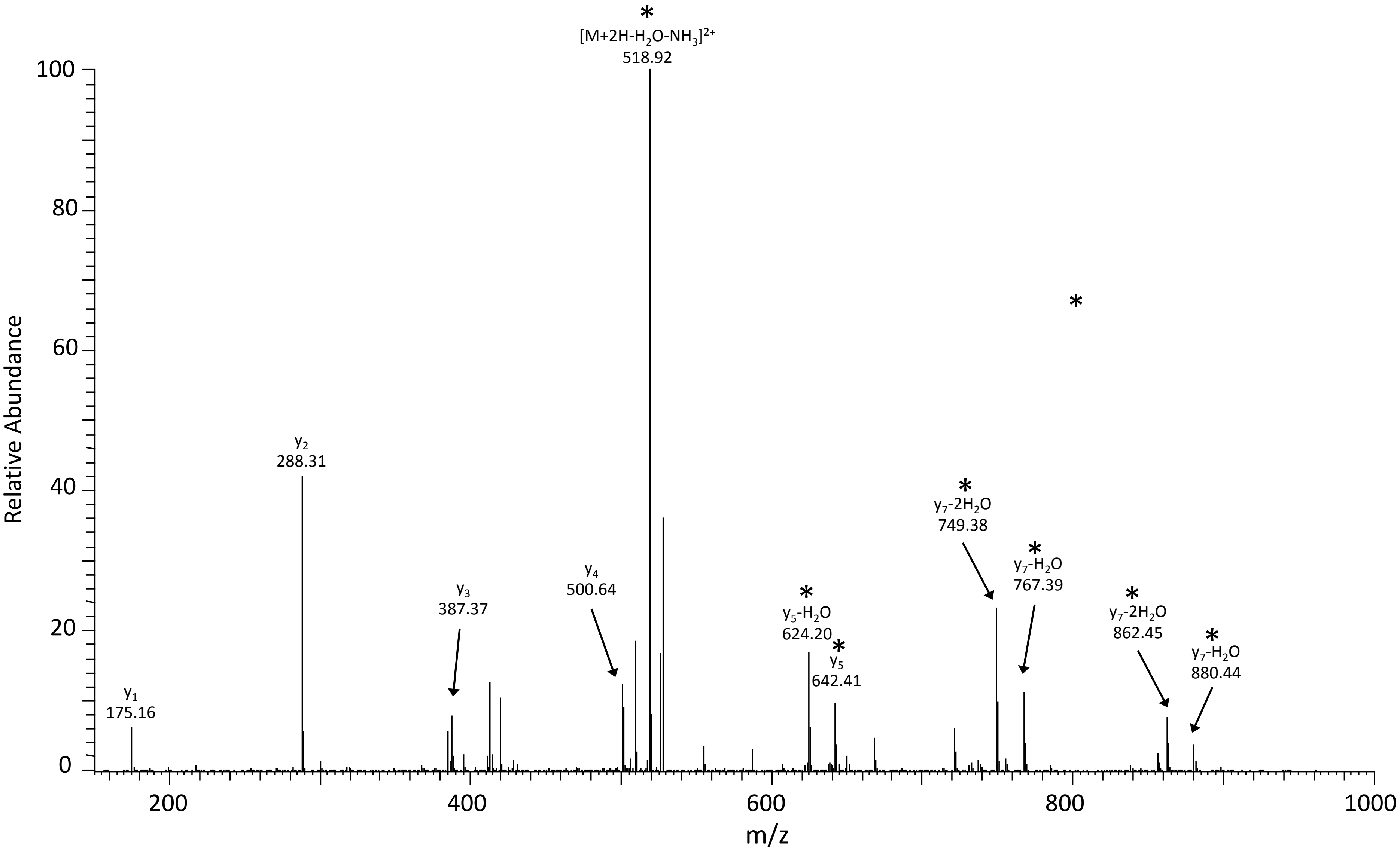

Sequence	Modifications	XCorr	Charge	m/z (Da)	MH+ (Da)	Δm (ppm)	t_r (min)	Enzyme
EstLHLVLRLRGG	S^{65}-Oxidation, T^{66}-Oxd'n	2.32	3	488.9490	1464.8325	2.91	28.46	LysC

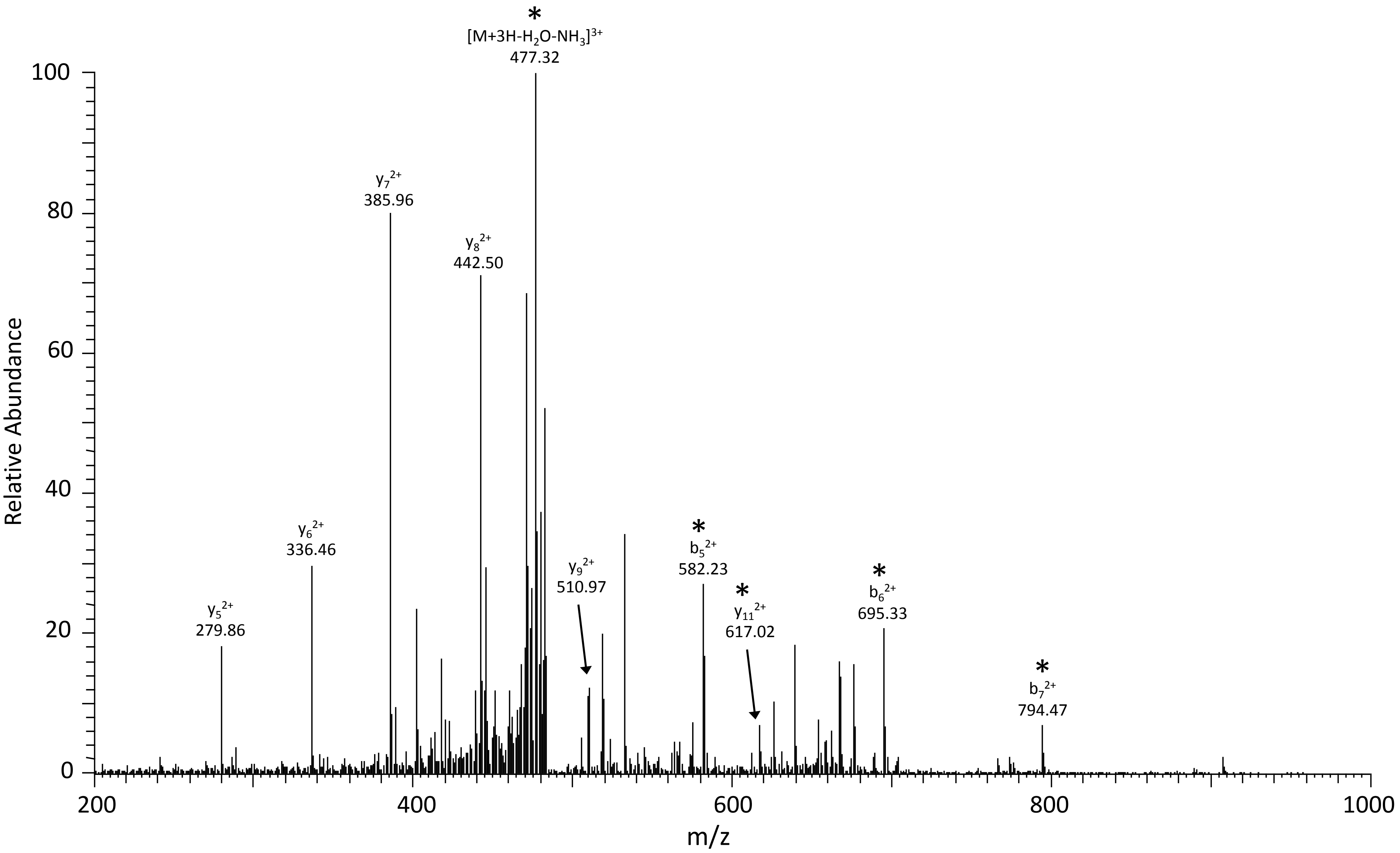

Sequence	Modifications	XCorr	Charge	m/z (Da)	MH+ (Da)	Δm (ppm)	t_r (min)	Enzyme
EVEPSDTIeNVKAKIQ	E^{24}-Decarboxylation	2.63	2	885.4650	1769.9227	-3.05	28.22	LysC

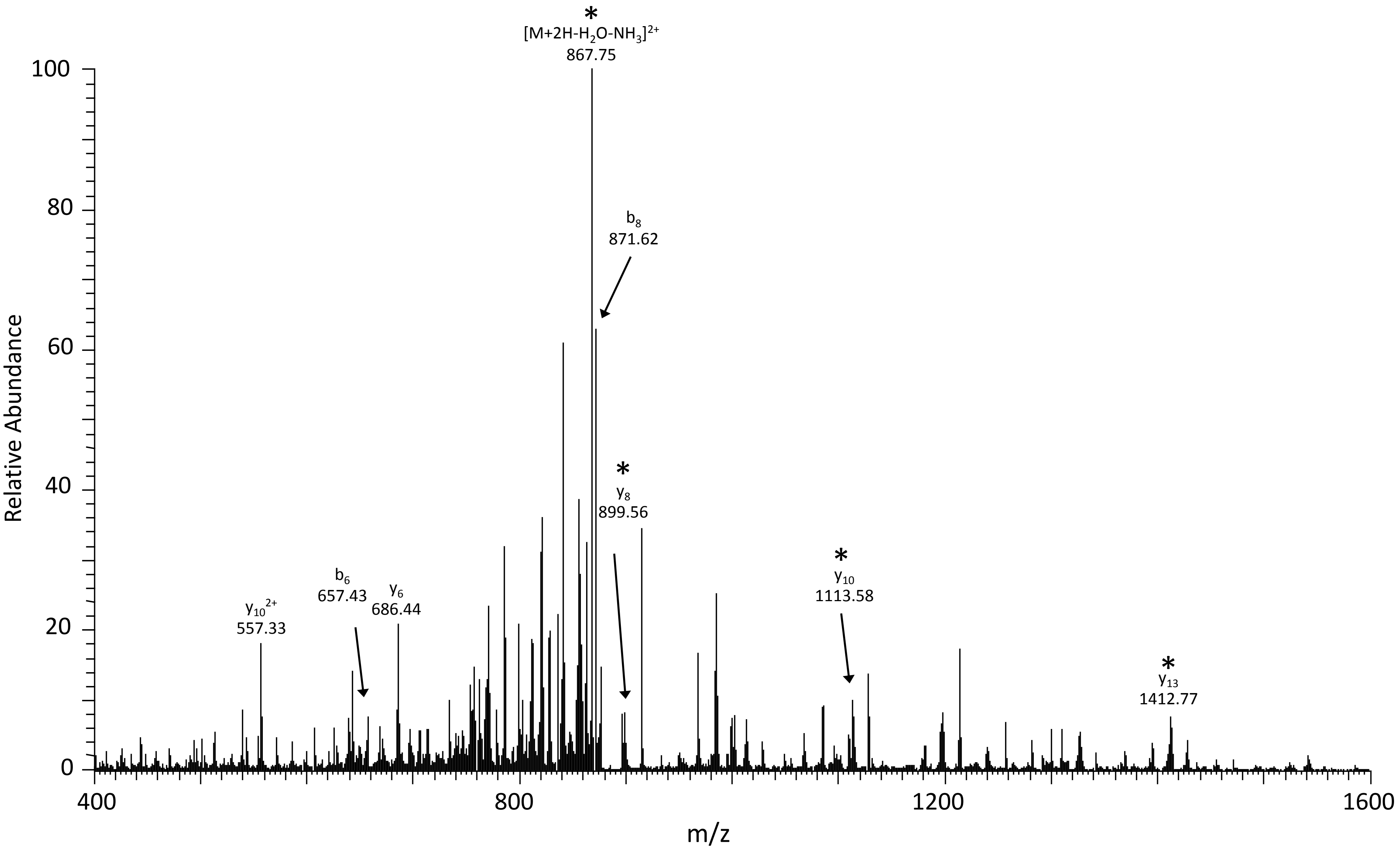

Sequence	Modifications	XCorr	Charge	m/z (Da)	MH+ (Da)	Δm (ppm)	t_r (min)	Enzyme
GkQLEDGR	K^{48}-Carbonylation	1.98	2	458.7275	916.4476	-0.78	14.51	Trypsin

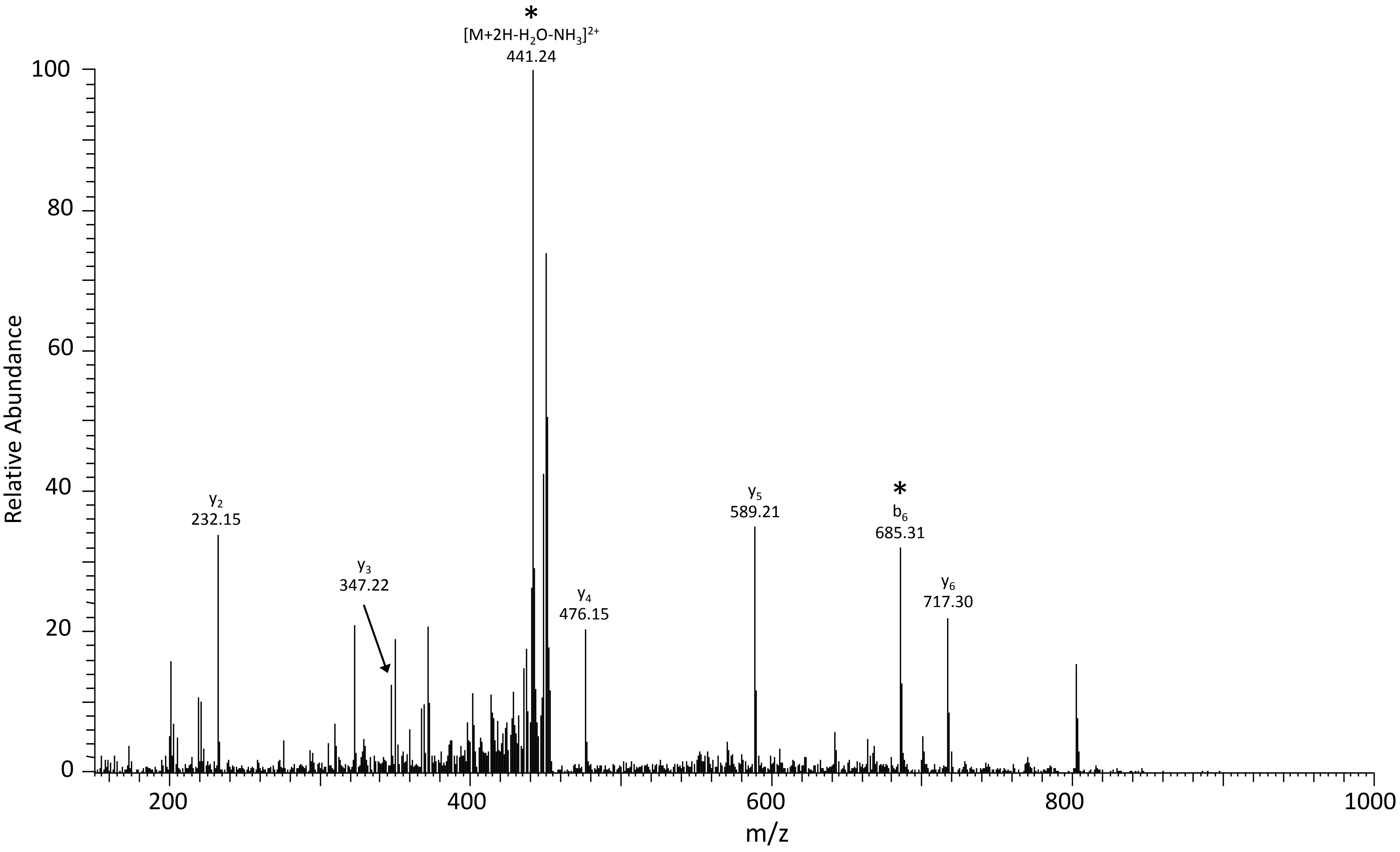

Sequence	Modifications	XCorr	Charge	m/z (Da)	MH+ (Da)	Δm (ppm)	t_r (min)	Enzyme
IQDKEGIPpDQQR	P^{38}-Dioxidation	3.67	2	778.3890	1555.7707	-0.30	19.16	Trypsin

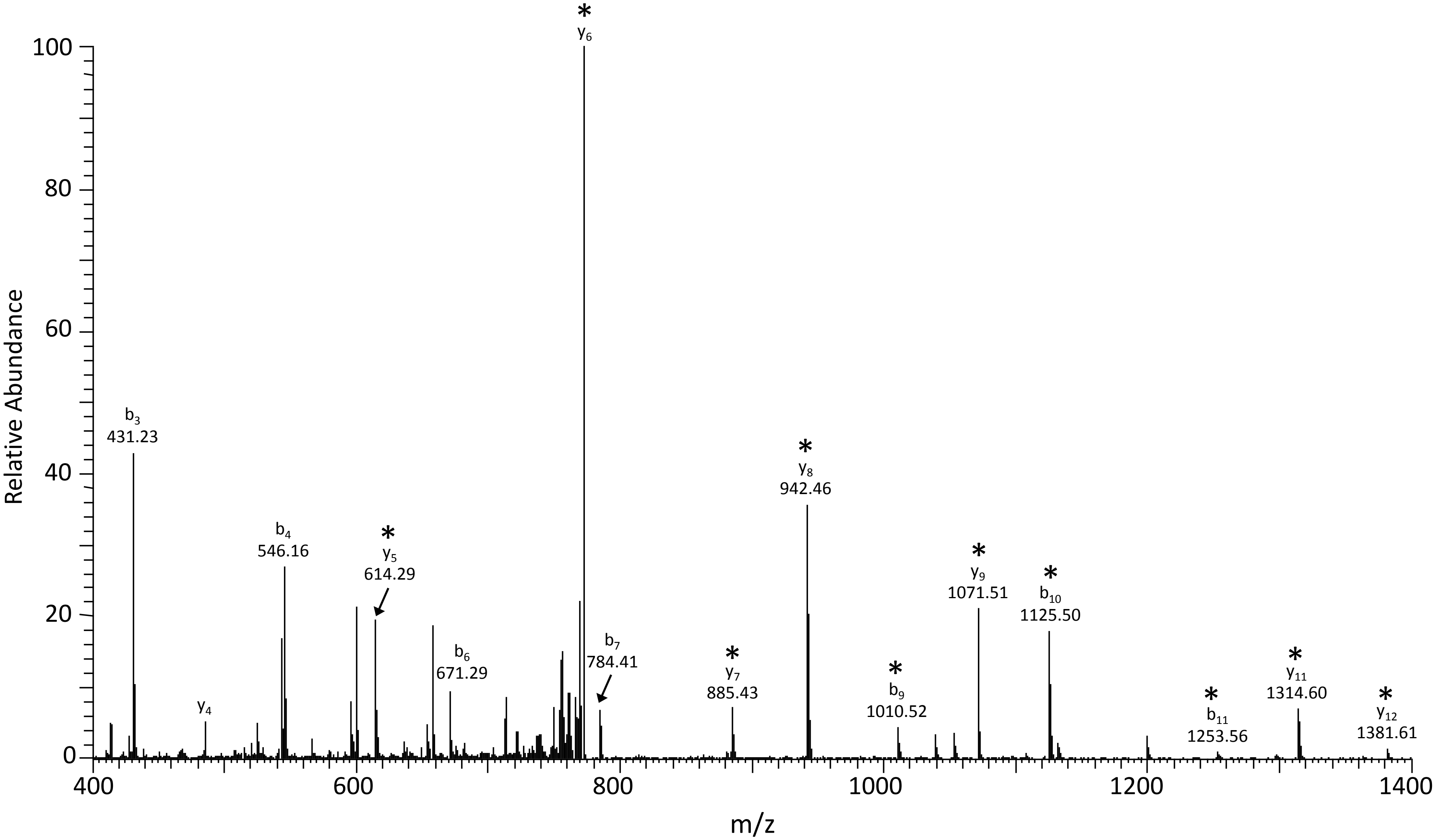

Sequence	Modifications	XCorr	Charge	m/z (Da)	MH+ (Da)	Δm (ppm)	t_r (min)	Enzyme
IQDKEGiPPDQQR	I^{36}-Carbonylation	2.55	3	513.2579	1537.7591	-0.98	14.30	Trypsin

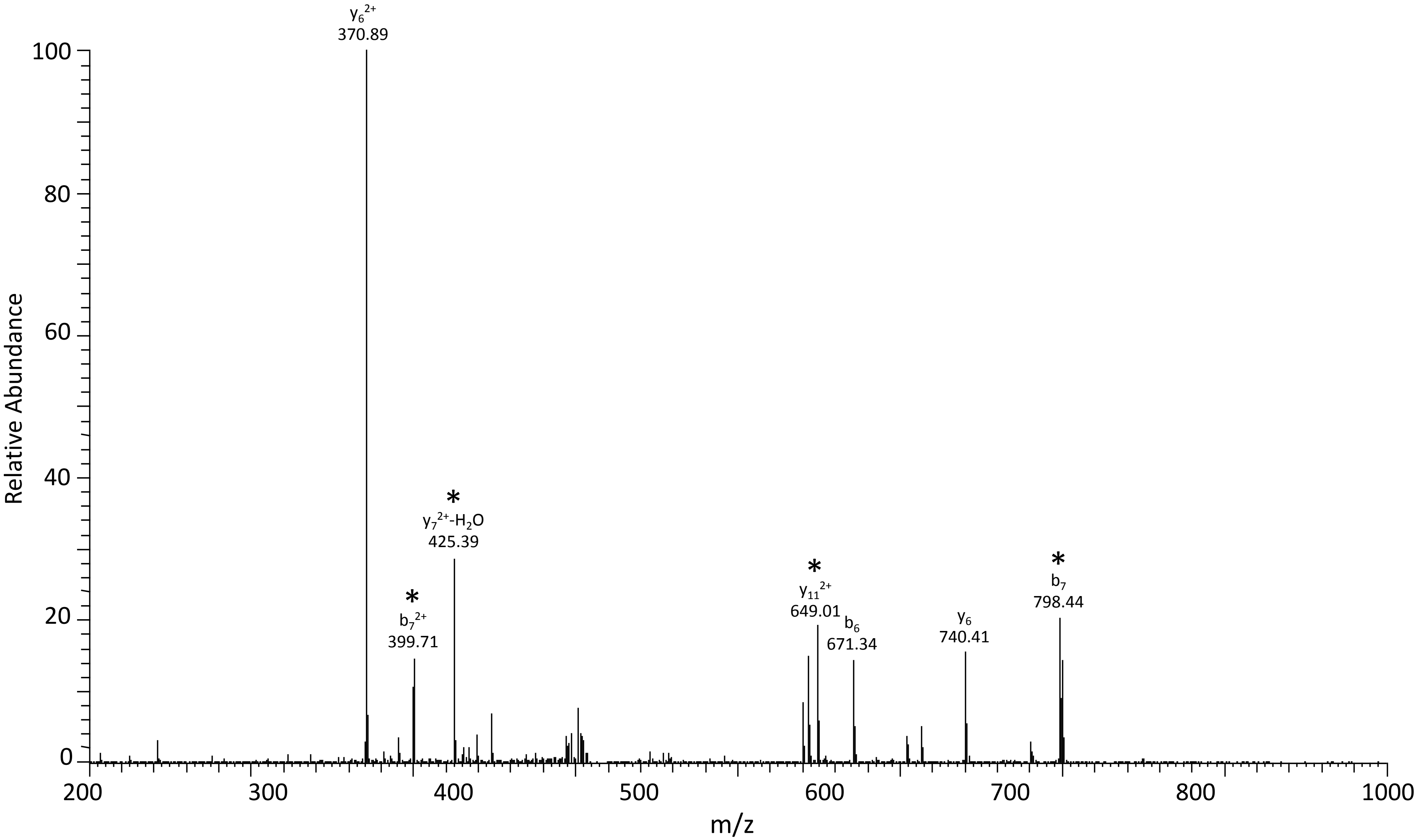

Sequence	Modifications	XCorr	Charge	m/z (Da)	MH+ (Da)	Δm (ppm)	t_r (min)	Enzyme
LIfAGKQLEDGR	F^{45}-Oxidation	2.29	3	454.9167	1362.7355	-1.58	16.12	Trypsin

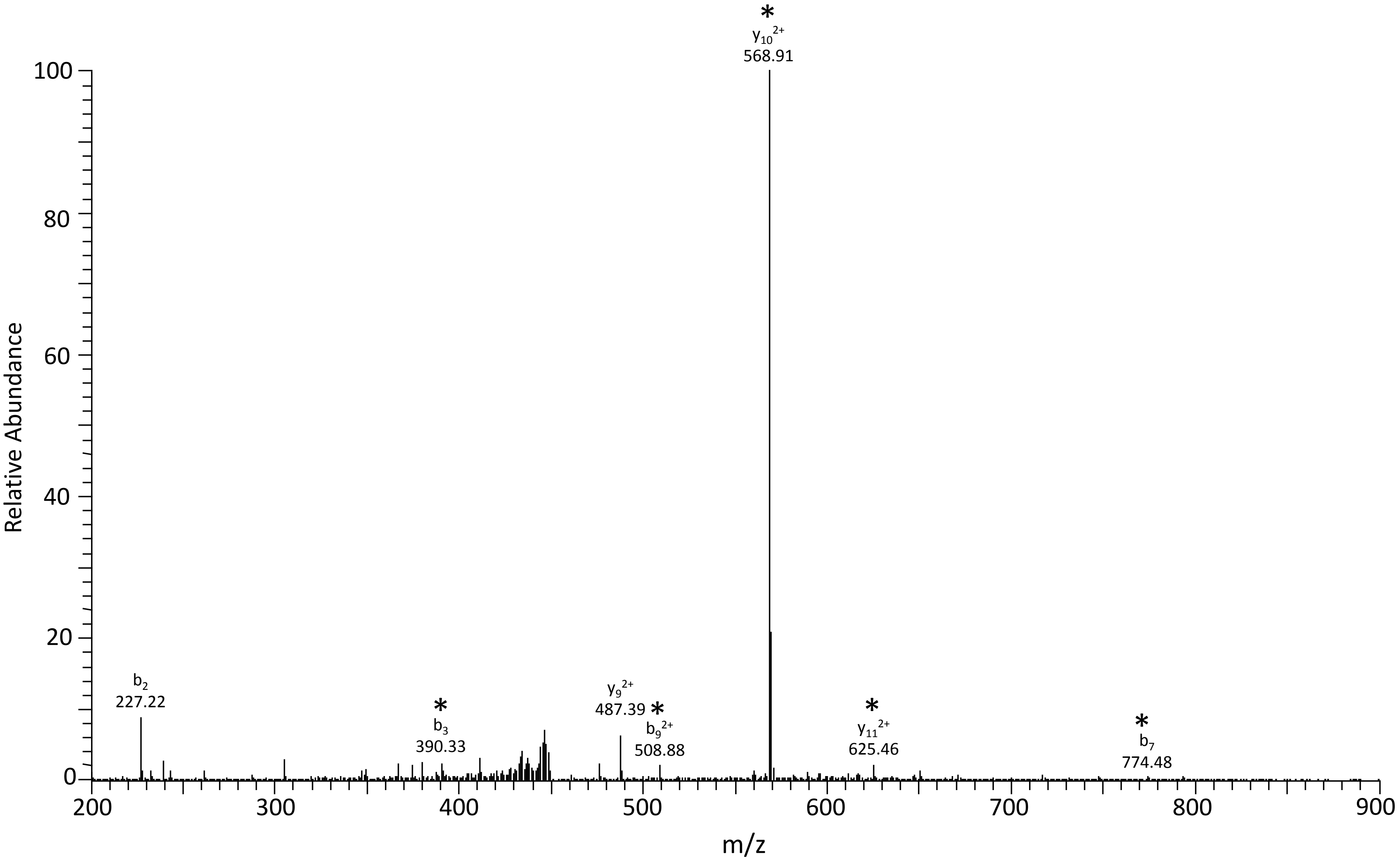

Sequence	Modifications	XCorr	Charge	m/z (Da)	MH+ (Da)	Δm (ppm)	t_r (min)	Enzyme
mQIfVK	M^1-Oxidation, F^4-Oxidation	2.16	2	399.2154	797.4235	1.10	21.18	LysC

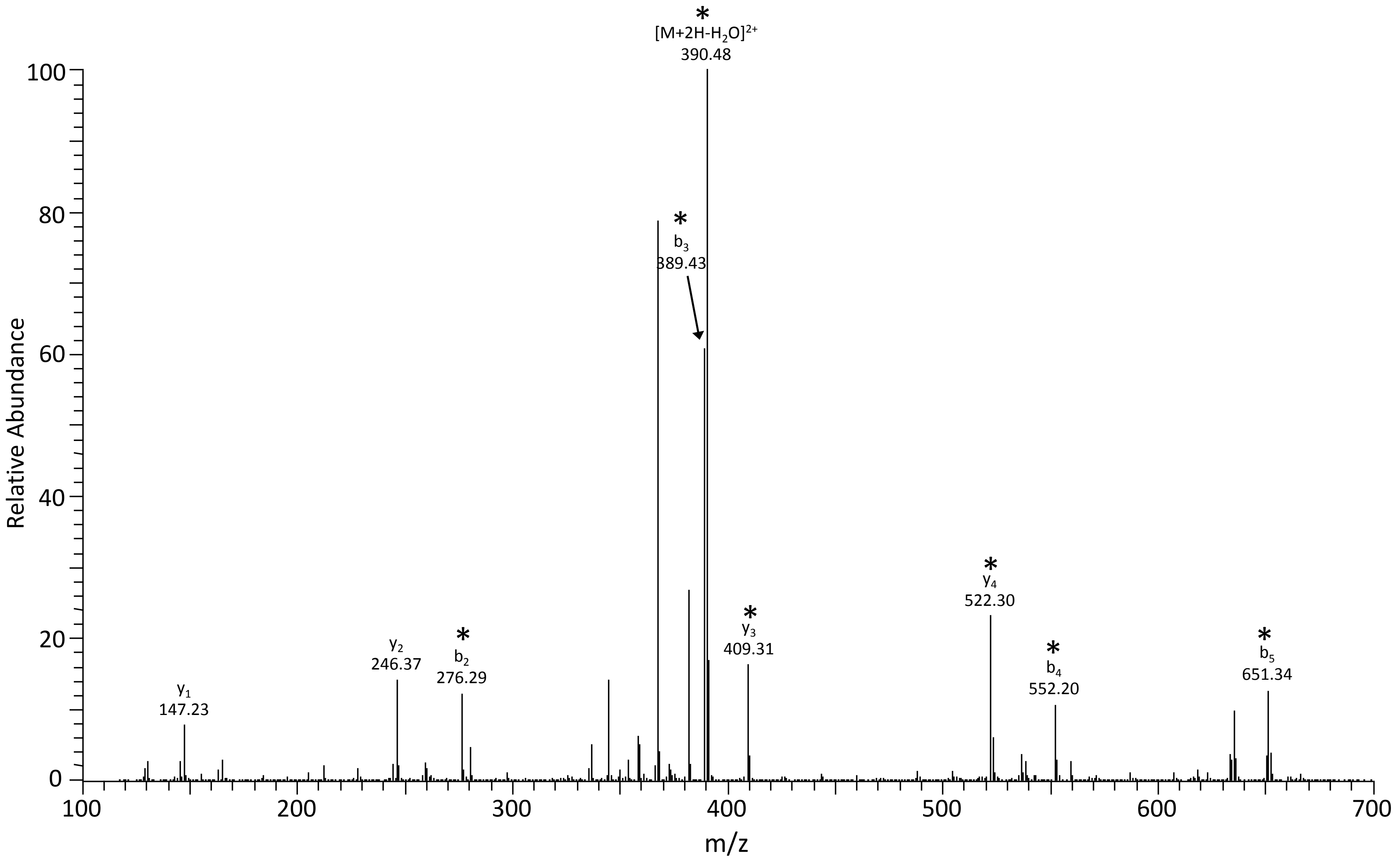

Sequence	Modifications	XCorr	Charge	m/z (Da)	MH+ (Da)	Δm (ppm)	t_r (min)	Enzyme
MQIfVK	F^4-Oxidation	1.97	2	391.2171	781.4269	-1.09	22.53	LysC

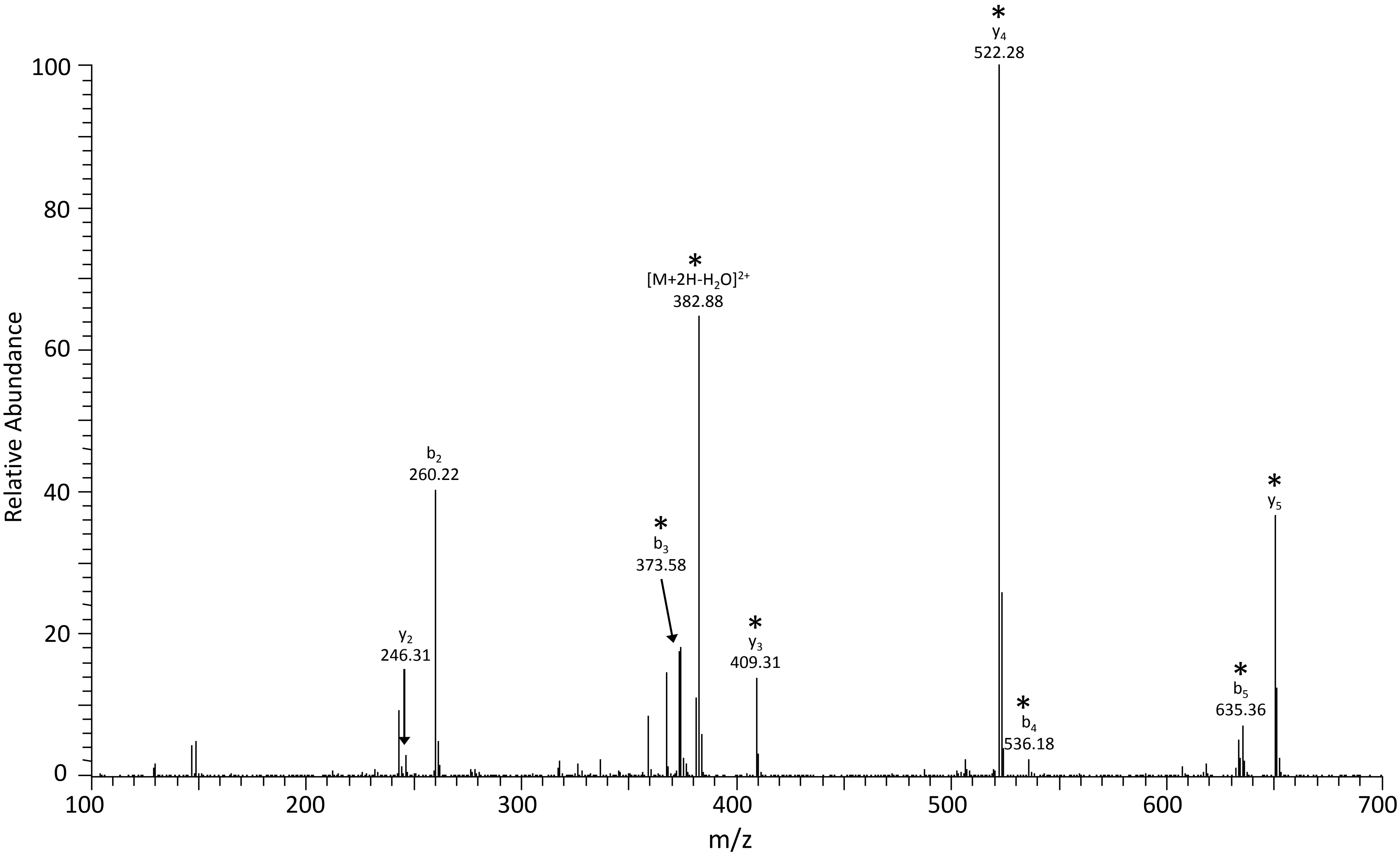

Sequence	Modifications	XCorr	Charge	m/z (Da)	MH+ (Da)	Δm (ppm)	t_r (min)	Enzyme
mQIFVK	M^1-Oxidation	1.77	1	781.4302	781.4302	3.23	22.04	GluC

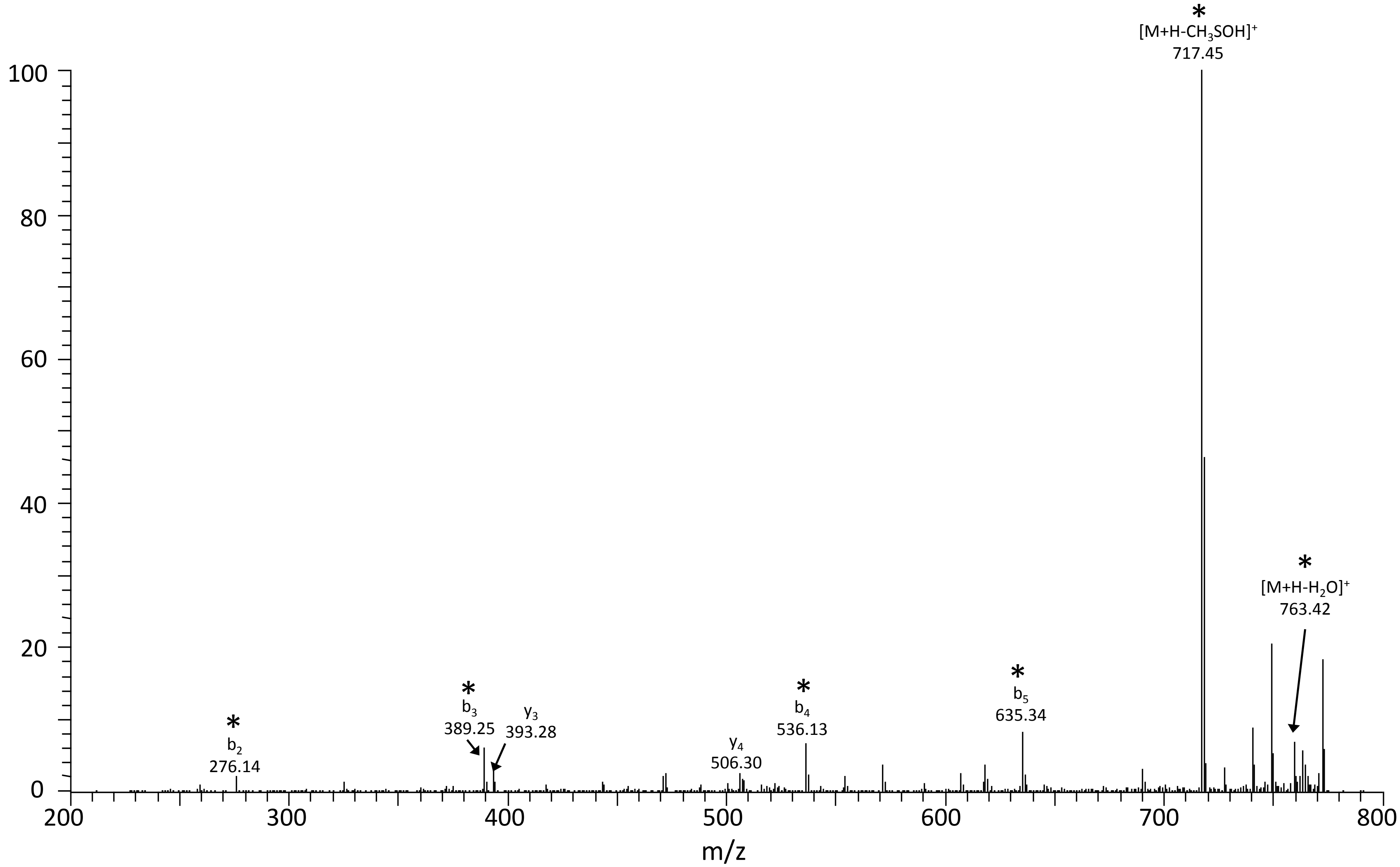

Sequence	Modifications	XCorr	Charge	m/z (Da)	MH+ (Da)	Δm (ppm)	t_r (min)	Enzyme
mQIfVK	M^1-Oxidation, F^4-Oxidation	2.13	2	399.2150	797.4228	0.18	21.01	Trypsin

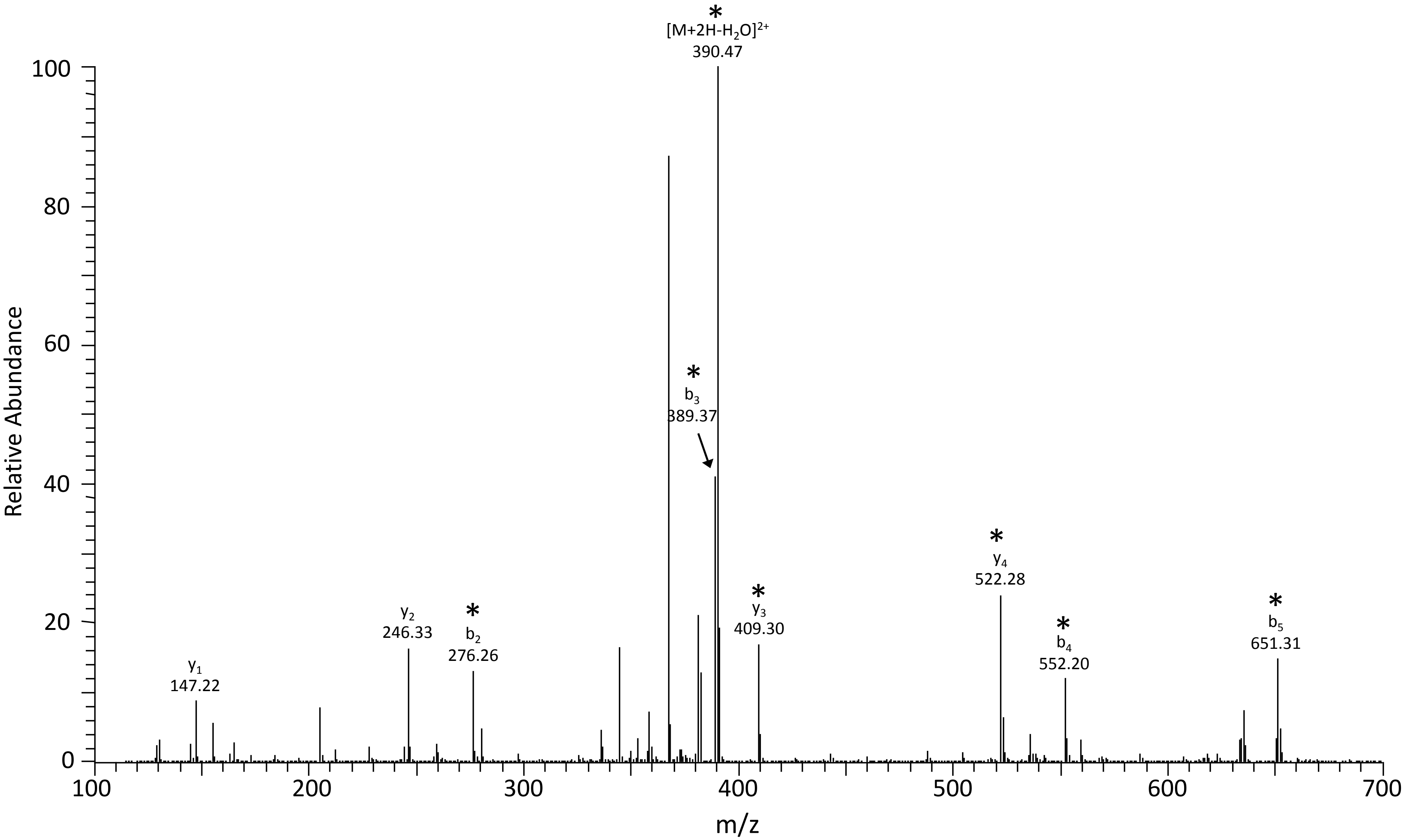

Sequence	Modifications	XCorr	Charge	m/z (Da)	MH+ (Da)	Δm (ppm)	t_r (min)	Enzyme
MQIfVK	F^4-Oxidation	1.79	2	391.2177	781.4282	0.63	22.09	Trypsin

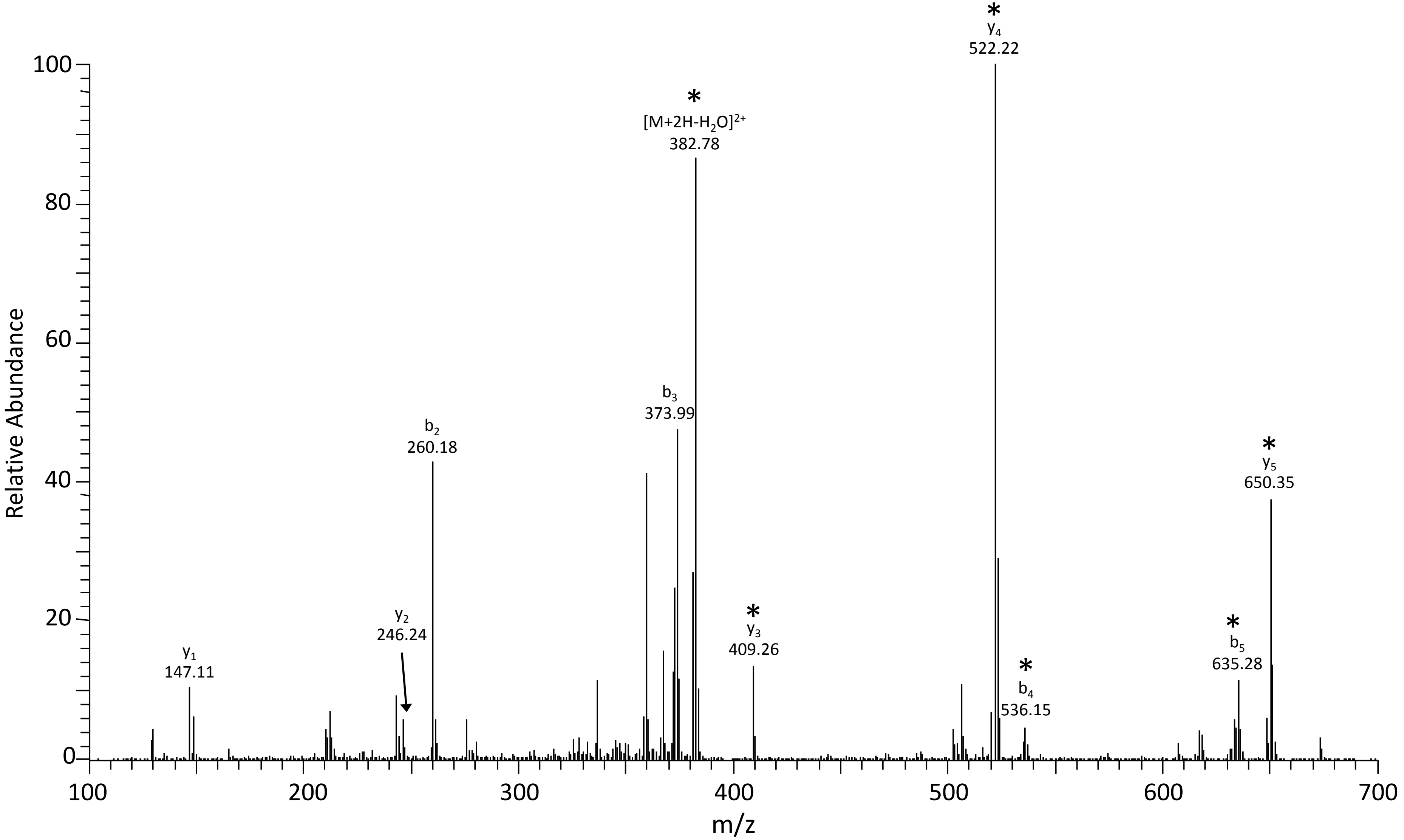

Sequence	Modifications	XCorr	Charge	m/z (Da)	MH+ (Da)	Δm (ppm)	t_r (min)	Enzyme
mQIFVK	M^1-Oxidation	1.72	1	781.4271	781.4271	-0.84	12.65	Trypsin

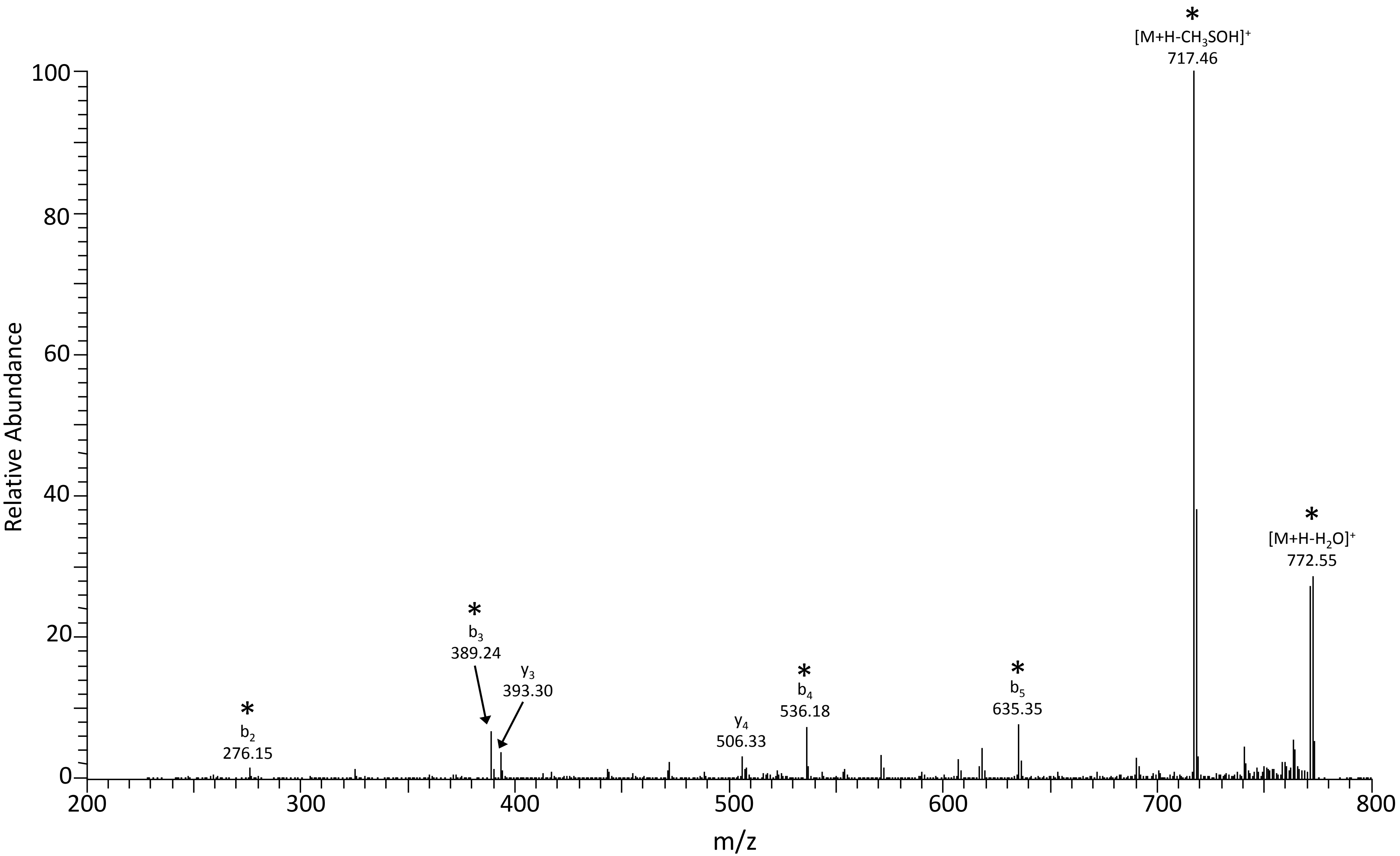

Sequence	Modifications	XCorr	Charge	m/z (Da)	MH+ (Da)	Δm (ppm)	t_r (min)	Enzyme
mQIFVK	M^1-Oxidation	1.96	2	391.2167	781.4261	-2.10	13.60	Trypsin

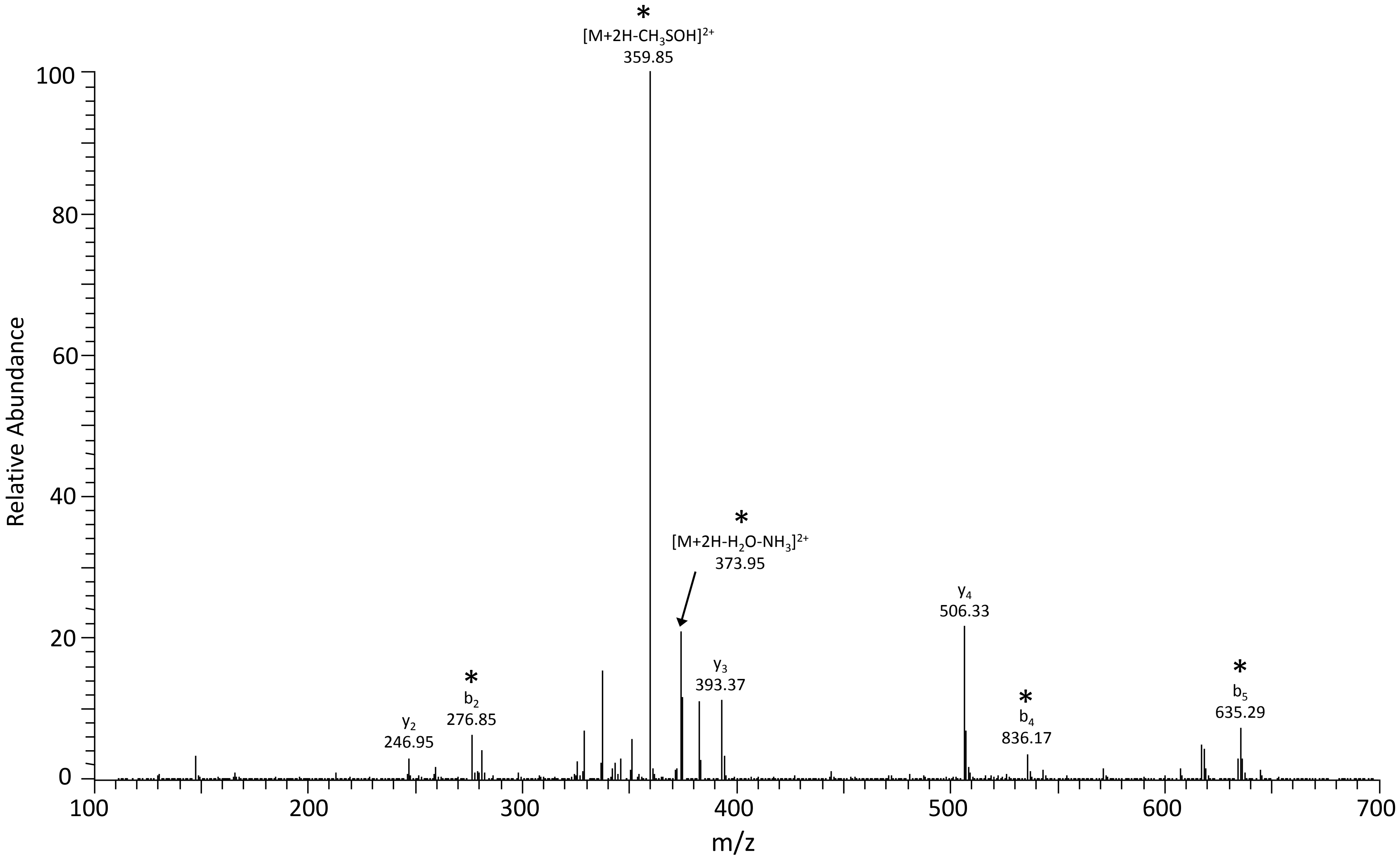

Sequence	Modifications	XCorr	Charge	m/z (Da)	MH+ (Da)	Δm (ppm)	t_r (min)	Enzyme
mQIFVK	M^1-Oxidation	1.76	1	781.4271	781.4271	-0.84	21.33	LysC

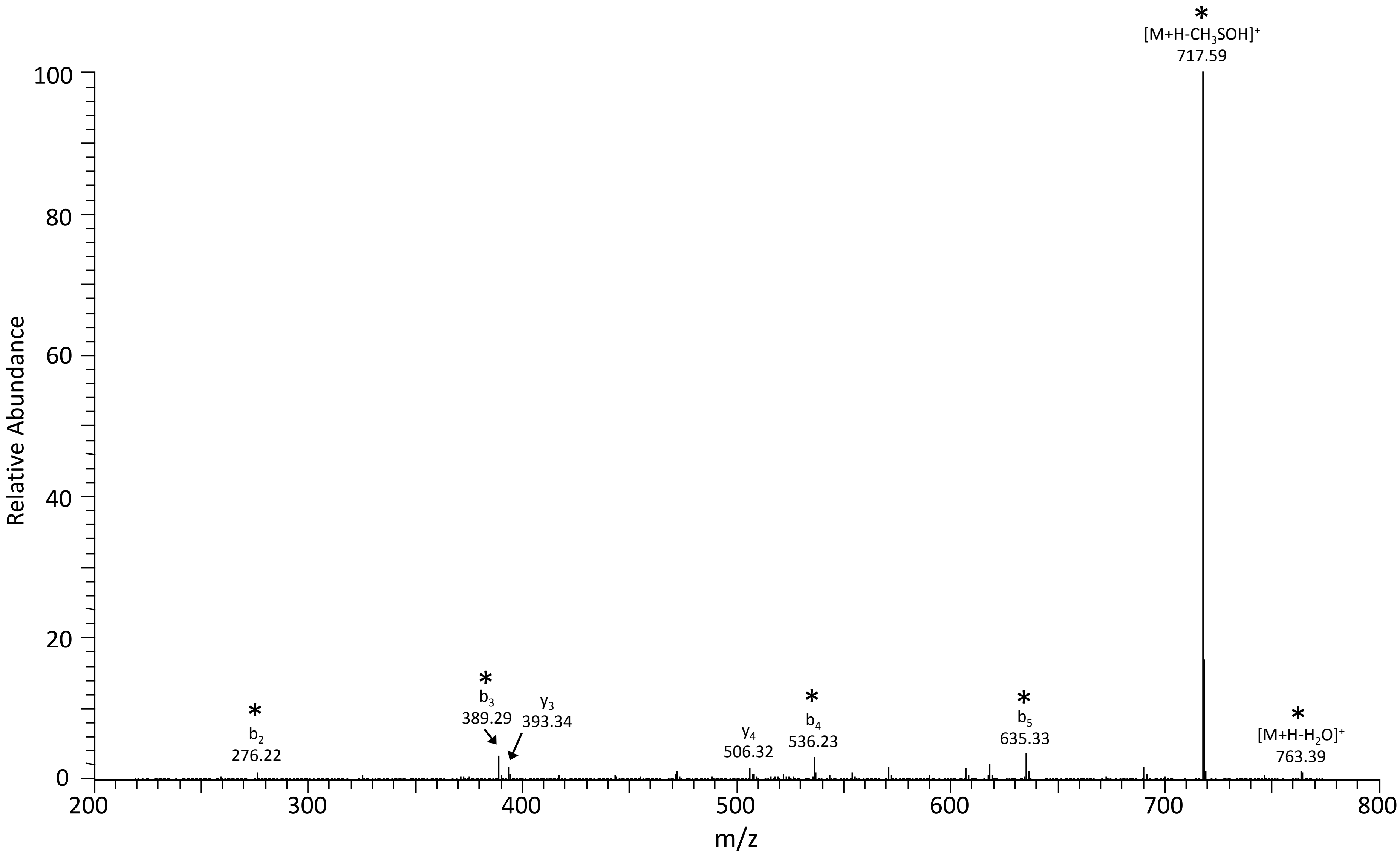

Sequence	Modifications	XCorr	Charge	m/z (Da)	MH+ (Da)	Δm (ppm)	t_r (min)	Enzyme
mQIFVKTL	M^1-Oxidation	2.84	2	498.2857	995.5641	4.63	25.08	GluC

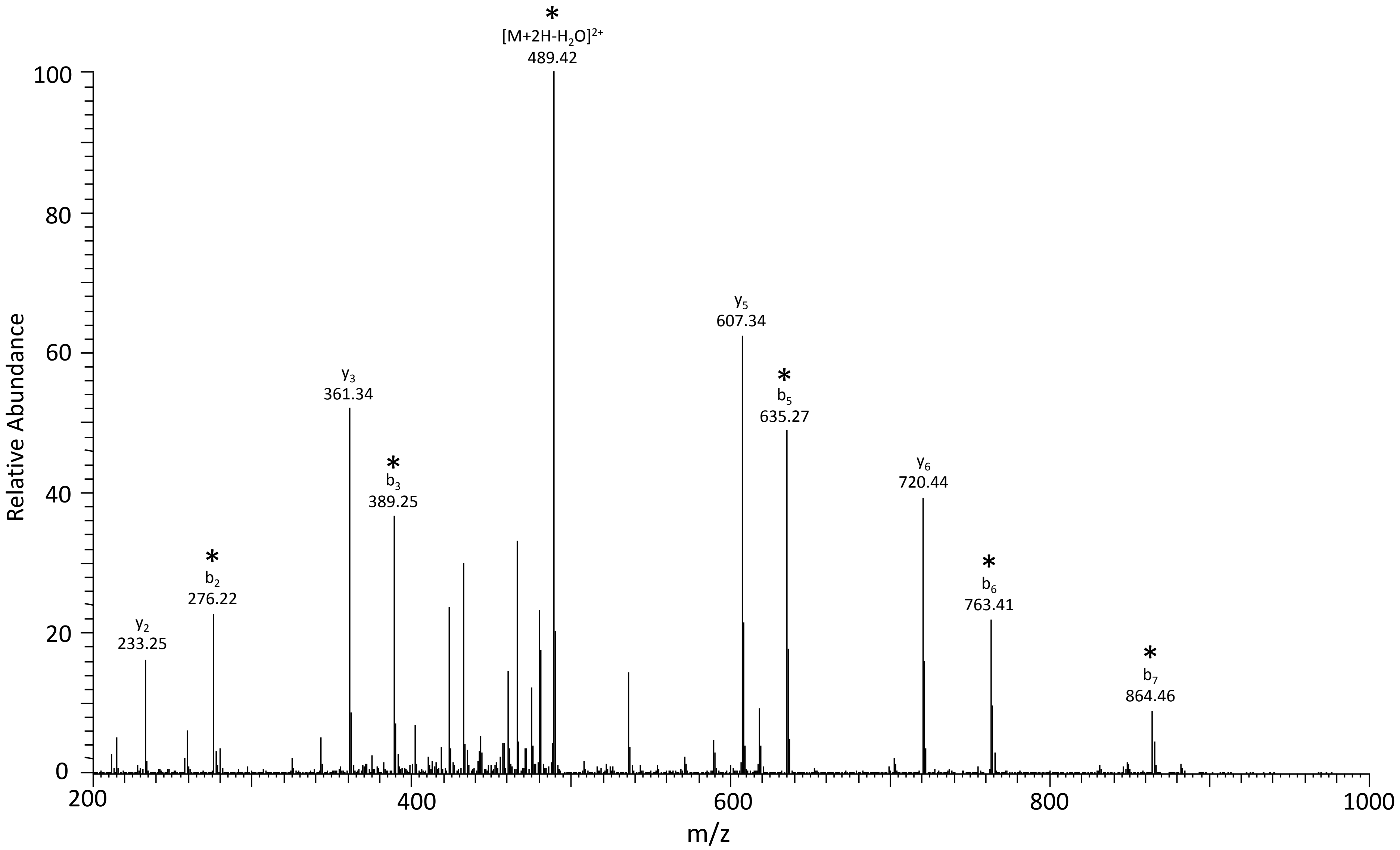

Sequence	Modifications	XCorr	Charge	m/z (Da)	MH+ (Da)	Δm (ppm)	t_r (min)	Enzyme
mQIFVKTLTGK	M^1-Oxidation	3.05	3	427.9143	1281.7283	3.66	23.55	GluC

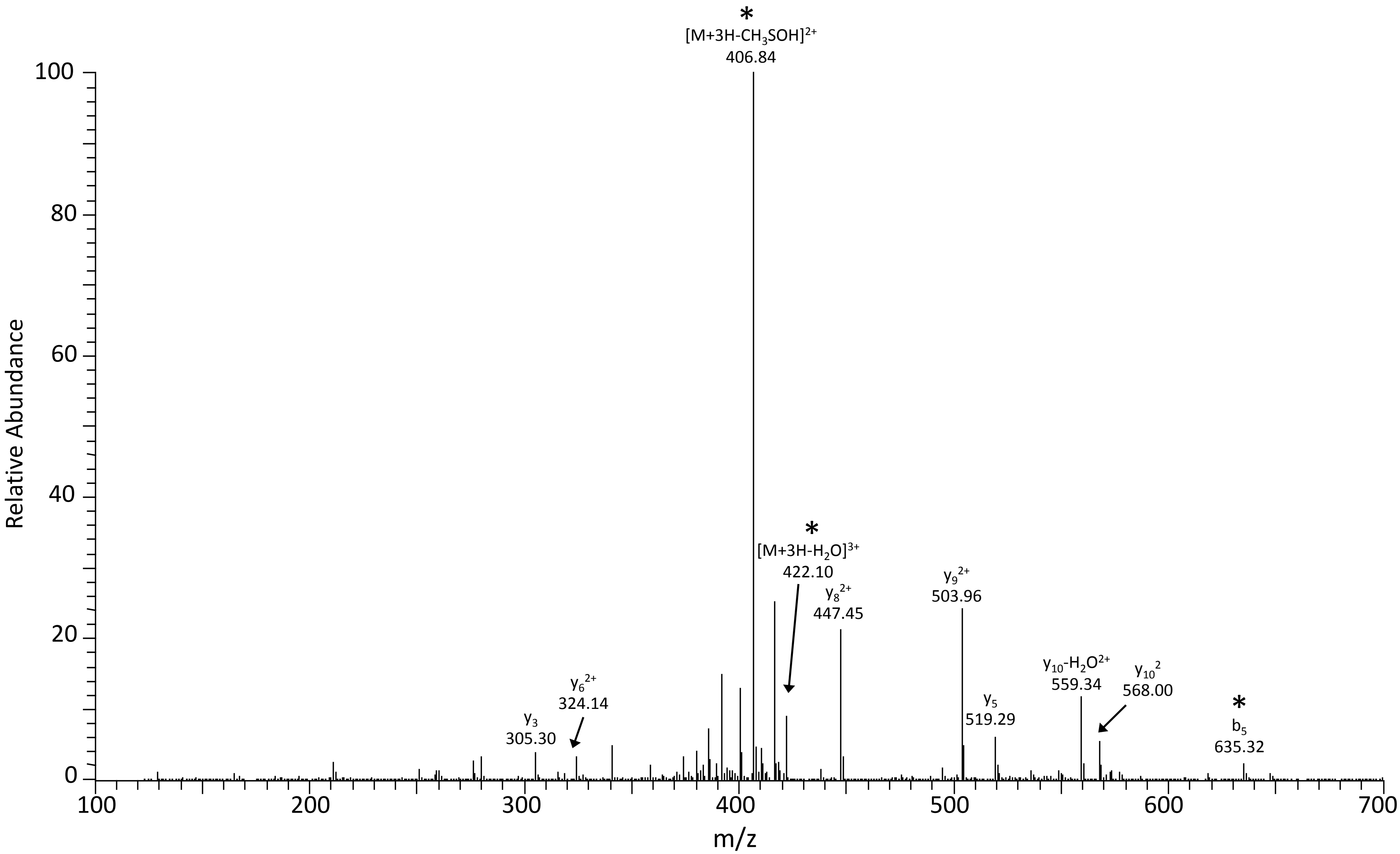

Sequence	Modifications	XCorr	Charge	m/z (Da)	MH+ (Da)	Δm (ppm)	t_r (min)	Enzyme
mQIFVKTLTGK	M^1-Oxidation	2.80	3	427.9117	1281.7207	-2.27	18.12	Trypsin

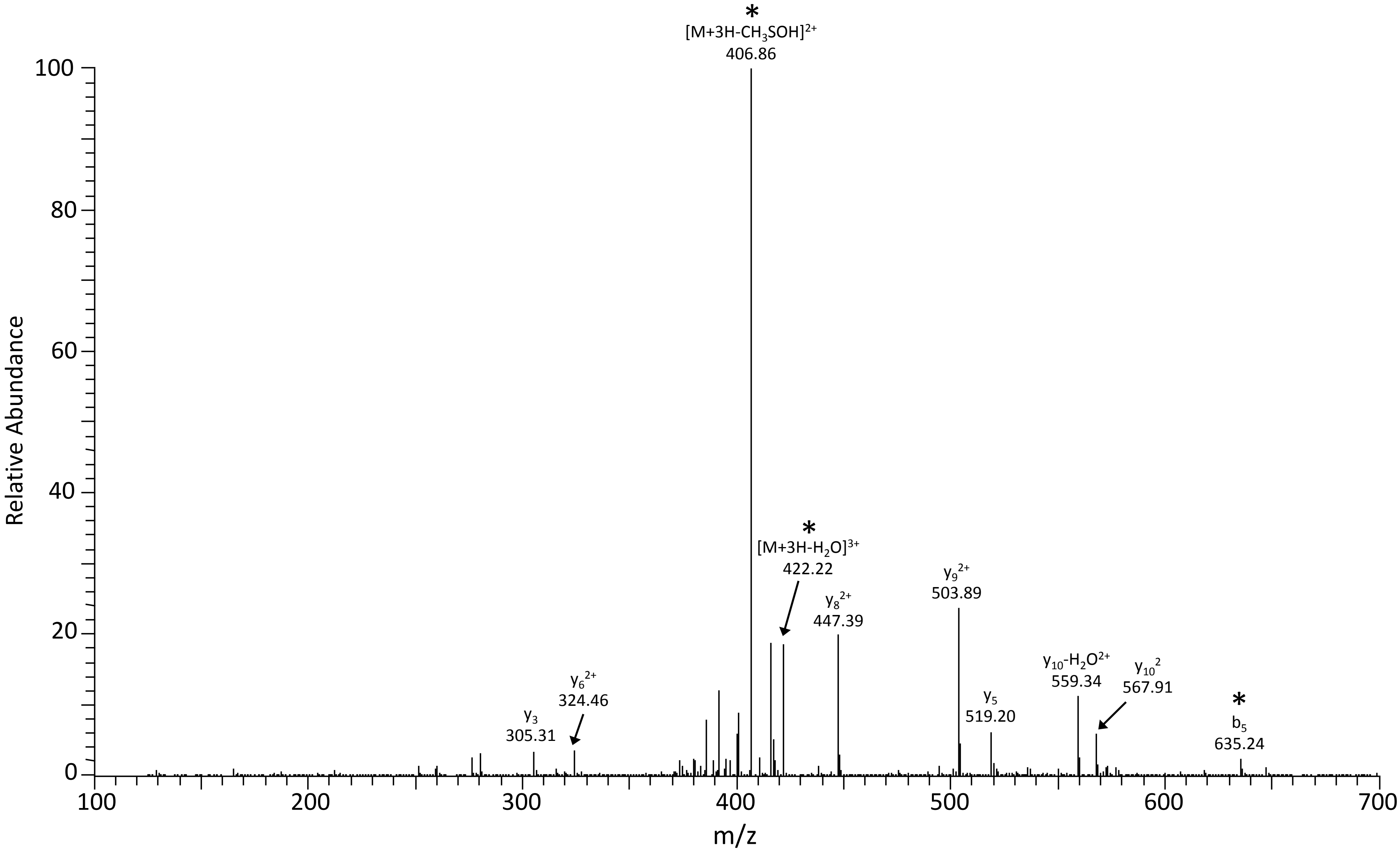

Sequence	Modifications	XCorr	Charge	m/z (Da)	MH+ (Da)	Δm (ppm)	t_r (min)	Enzyme
mQIFVKTLTGKTITLEVEPSDTIENVKAK	M^1-Oxidation	5.18	4	813.1945	3249.7560	-2.84	22.06	Trypsin

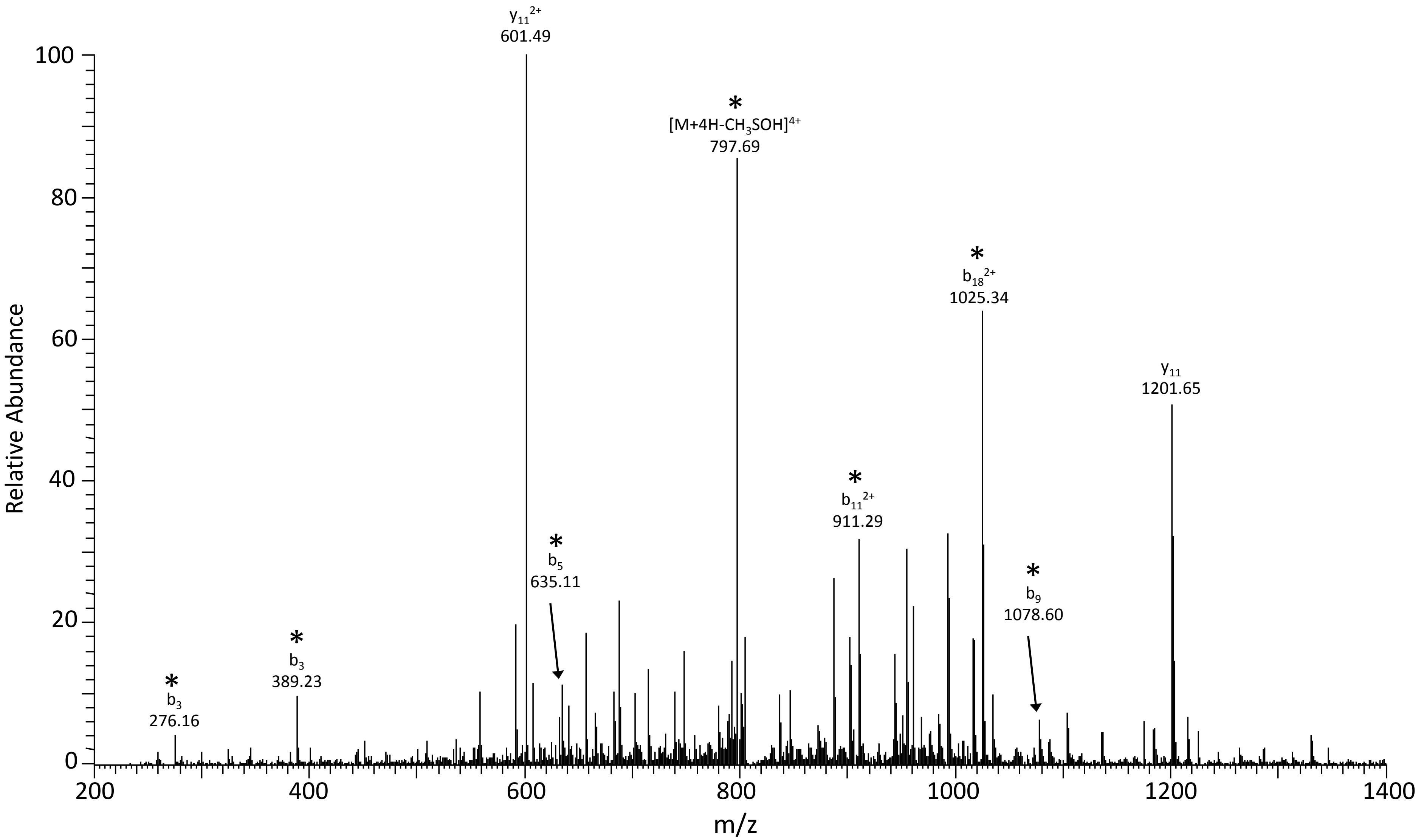

Sequence	Modifications	XCorr	Charge	m/z (Da)	MH+ (Da)	Δm (ppm)	t_r (min)	Enzyme
NVKAKIQDkeG	K^{33}-Oxidation; E^{34}-Decarboxylation	3.10	2	608.3413	1215.6752	4.92	18.97	GluC

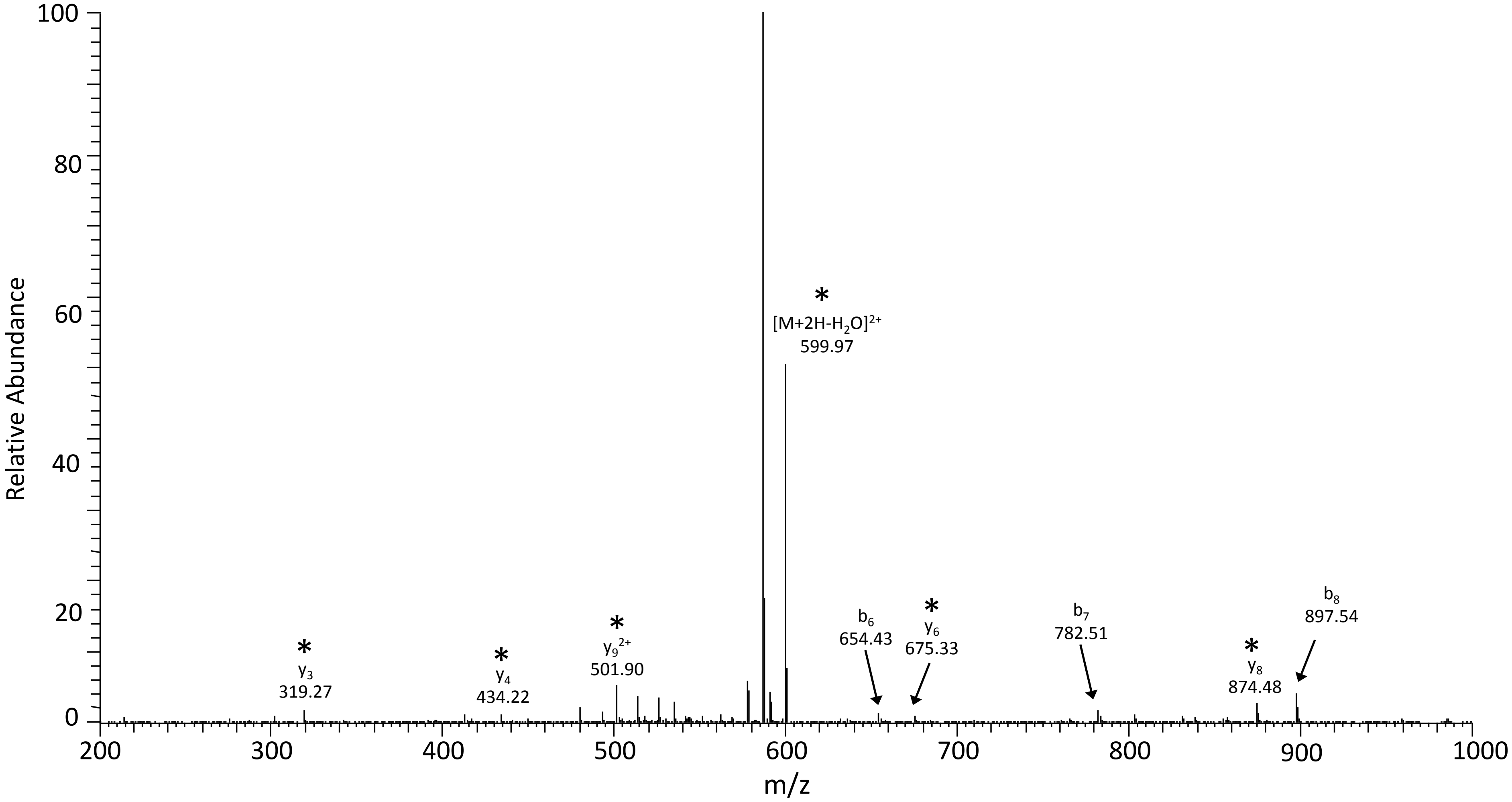

Sequence	Modifications	XCorr	Charge	m/z (Da)	MH+ (Da)	Δm (ppm)	t_r (min)	Enzyme
NVKAKIQDKEGIPp	P^{38}-Dioxidation	2.54	3	523.6282	1568.8702	3.71	20.44	GluC

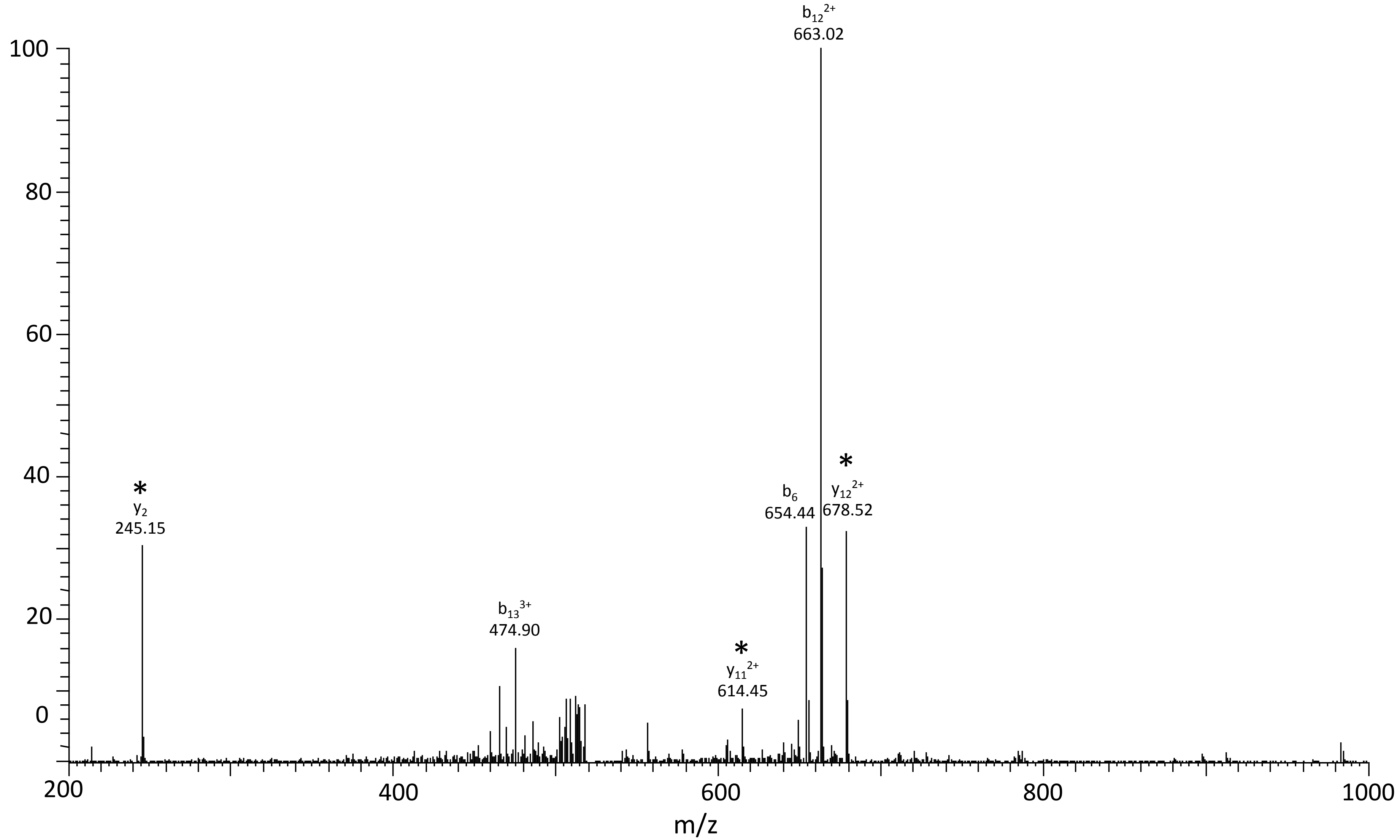

Sequence	Modifications	XCorr	Charge	m/z (Da)	MH+ (Da)	Δm (ppm)	t_r (min)	Enzyme
QLEDGrTLSDYNIQK	R^{54}-GluSA	2.34	2	868.9171	1736.8270	-3.94	19.35	Trypsin

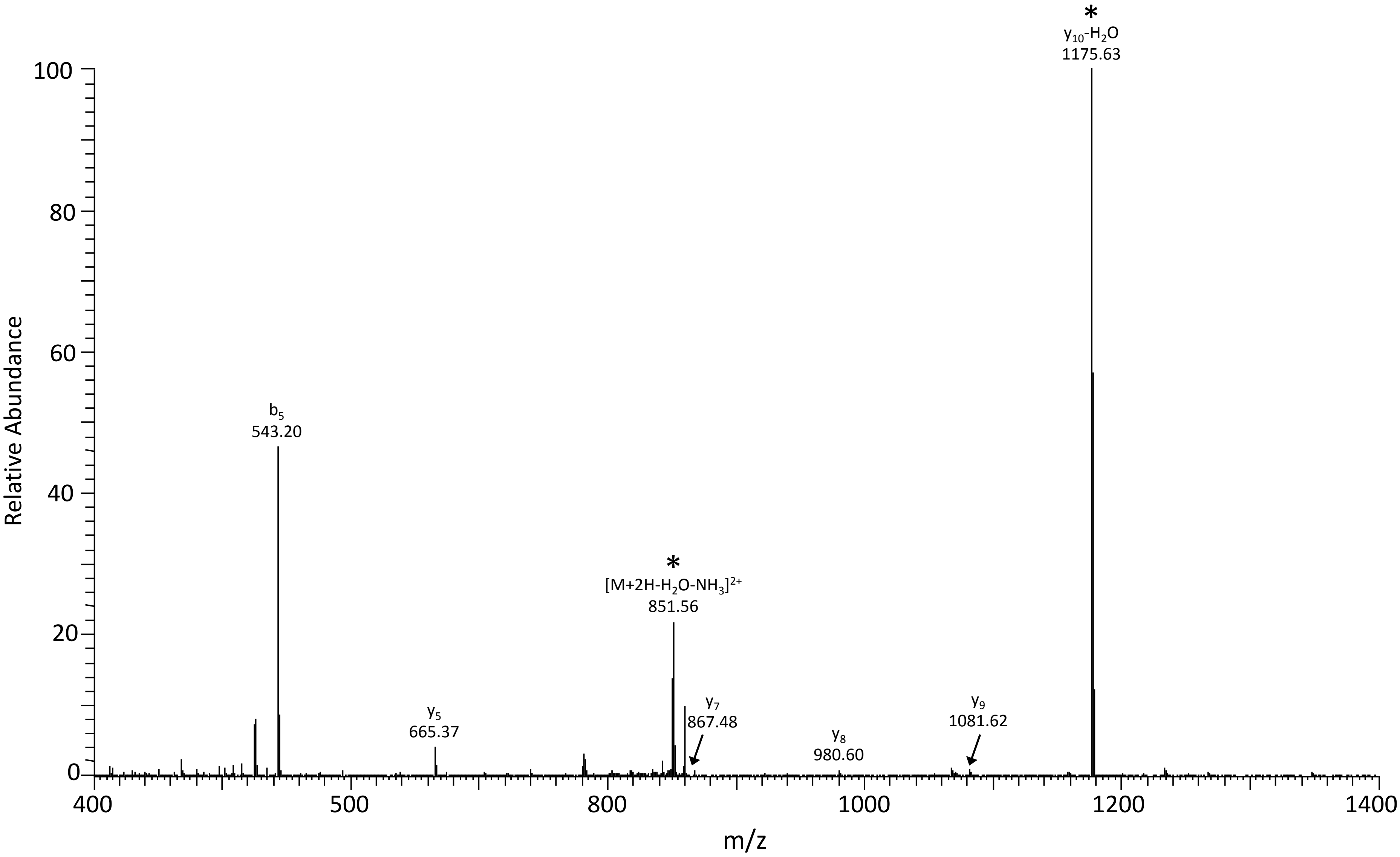

Sequence	Modifications	XCorr	Charge	m/z (Da)	MH+ (Da)	Δm (ppm)	t_r (min)	Enzyme
tITLEVEPSDTIENVK	T^{12}-Oxd'n	3.14	2	893.4609	1785.9145	1.54	25.96	LysC

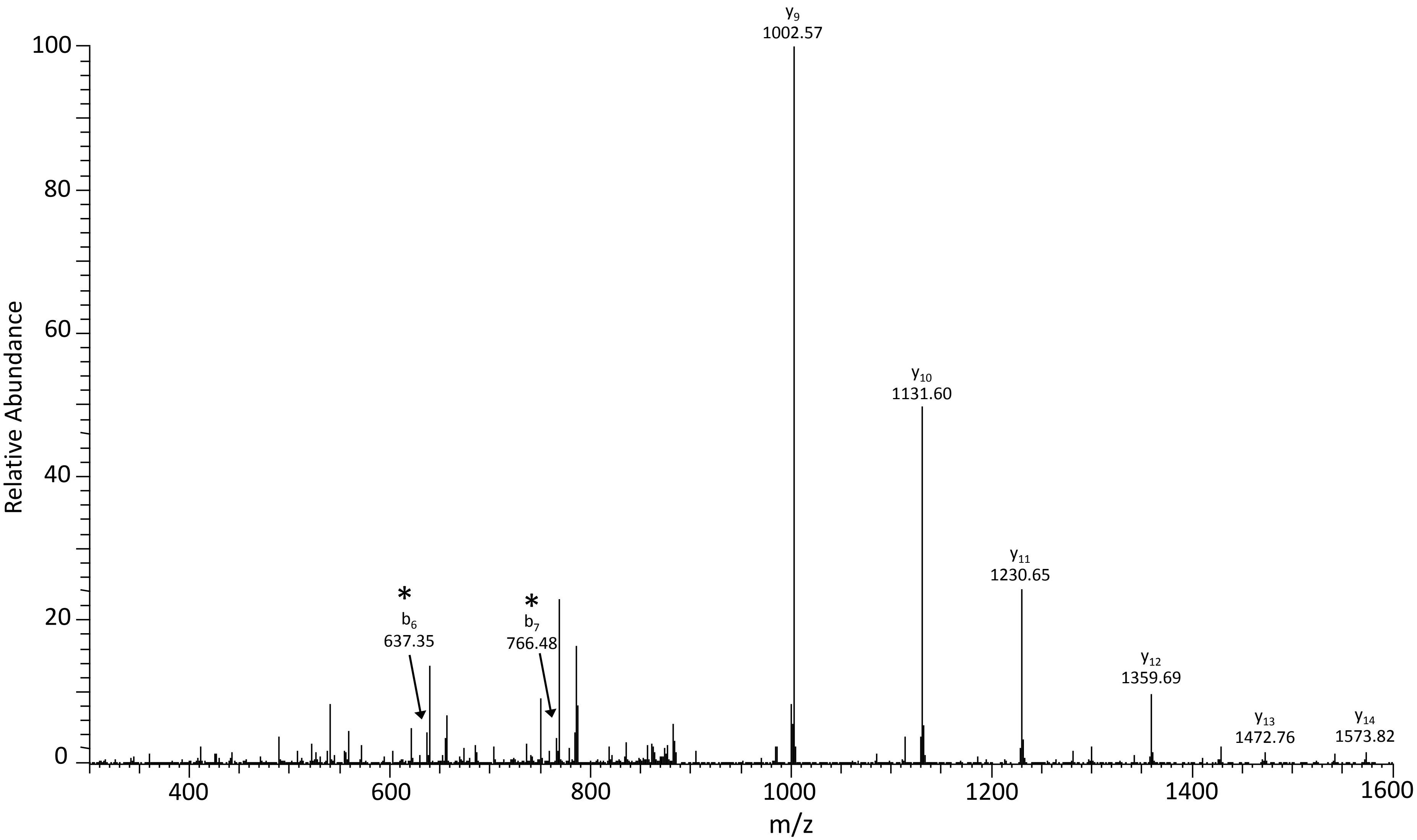

Sequence	Modifications	XCorr	Charge	m/z (Da)	MH+ (Da)	Δm (ppm)	t_r (min)	Enzyme
TITlEVEPSDTIENVK	L^{15}-Oxidation	3.44	2	902.4661	1803.9249	1.42	24.32	Trypsin

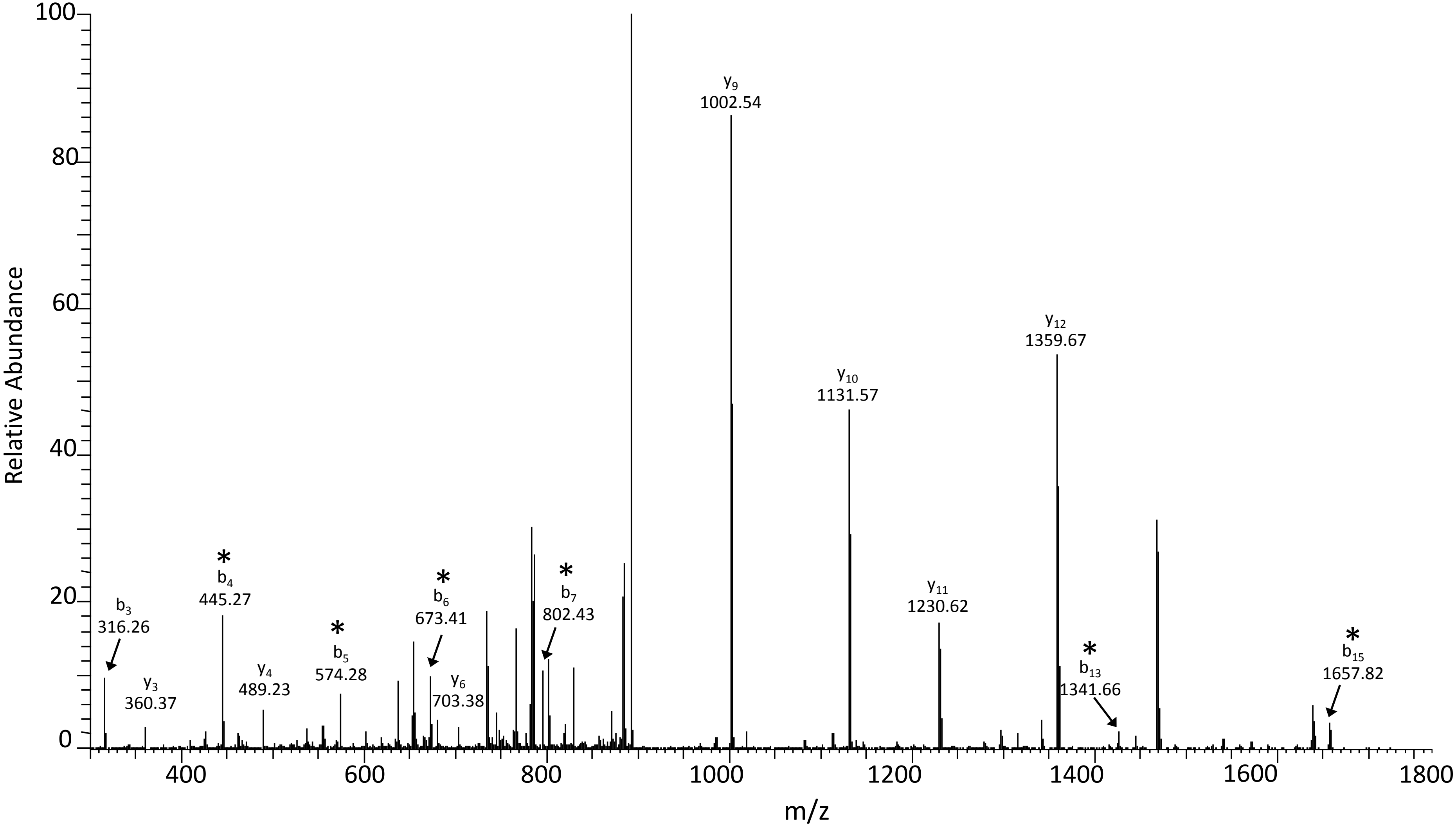

Sequence	Modifications	XCorr	Charge	m/z (Da)	MH+ (Da)	Δm (ppm)	t_r (min)	Enzyme
TITLEVEPsDTIENVK	S^{20}-Carbonylation	2.83	2	901.4577	1801.9081	0.83	24.64	Trypsin

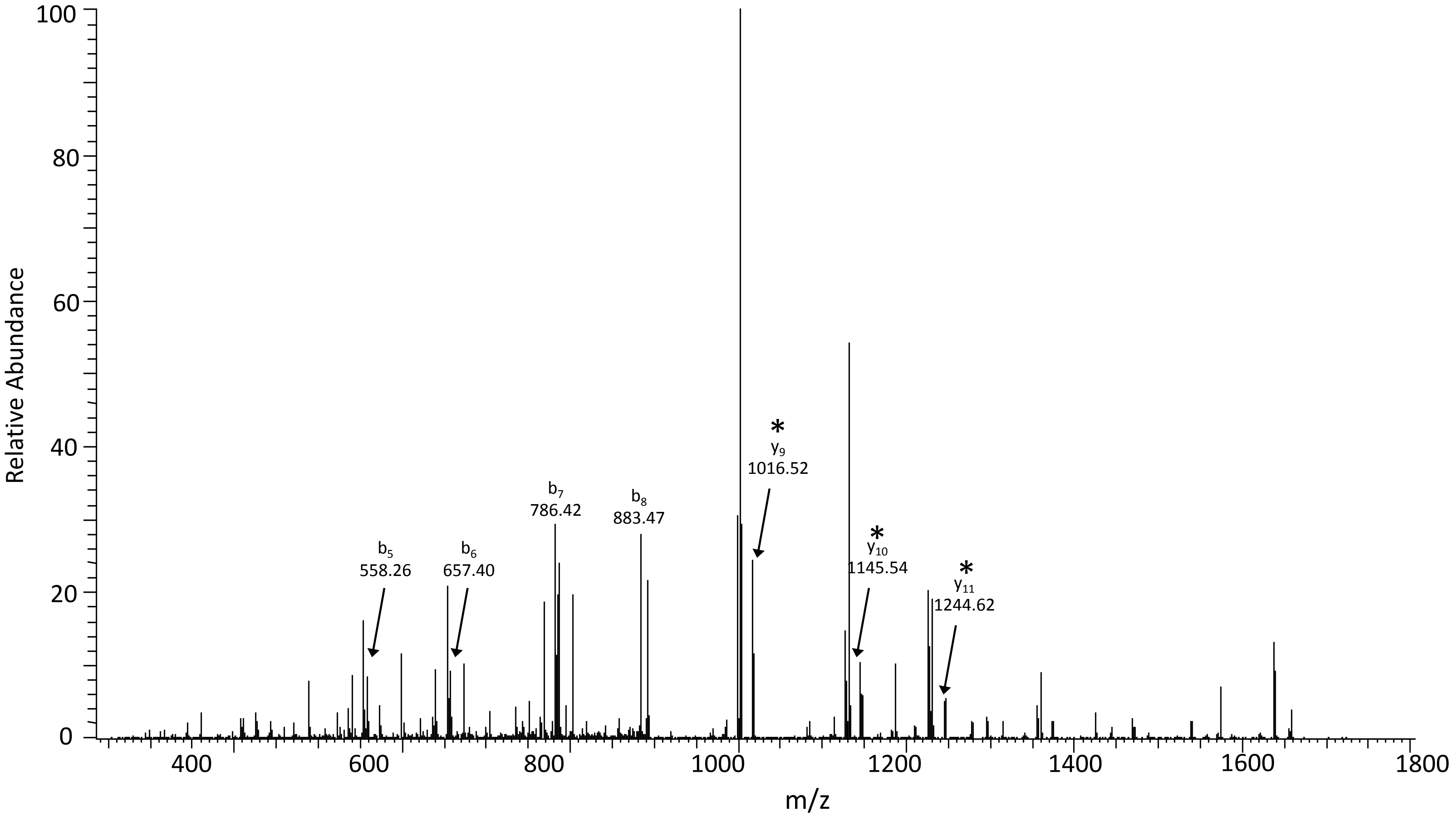

Sequence	Modifications	XCorr	Charge	m/z (Da)	MH+ (Da)	Δm (ppm)	t_r (min)	Enzyme
TITLEVePSDTIENVK	E^{18}-Decarboxylation	2.03	2	879.4589	1757.9105	-3.62	22.07	Trypsin

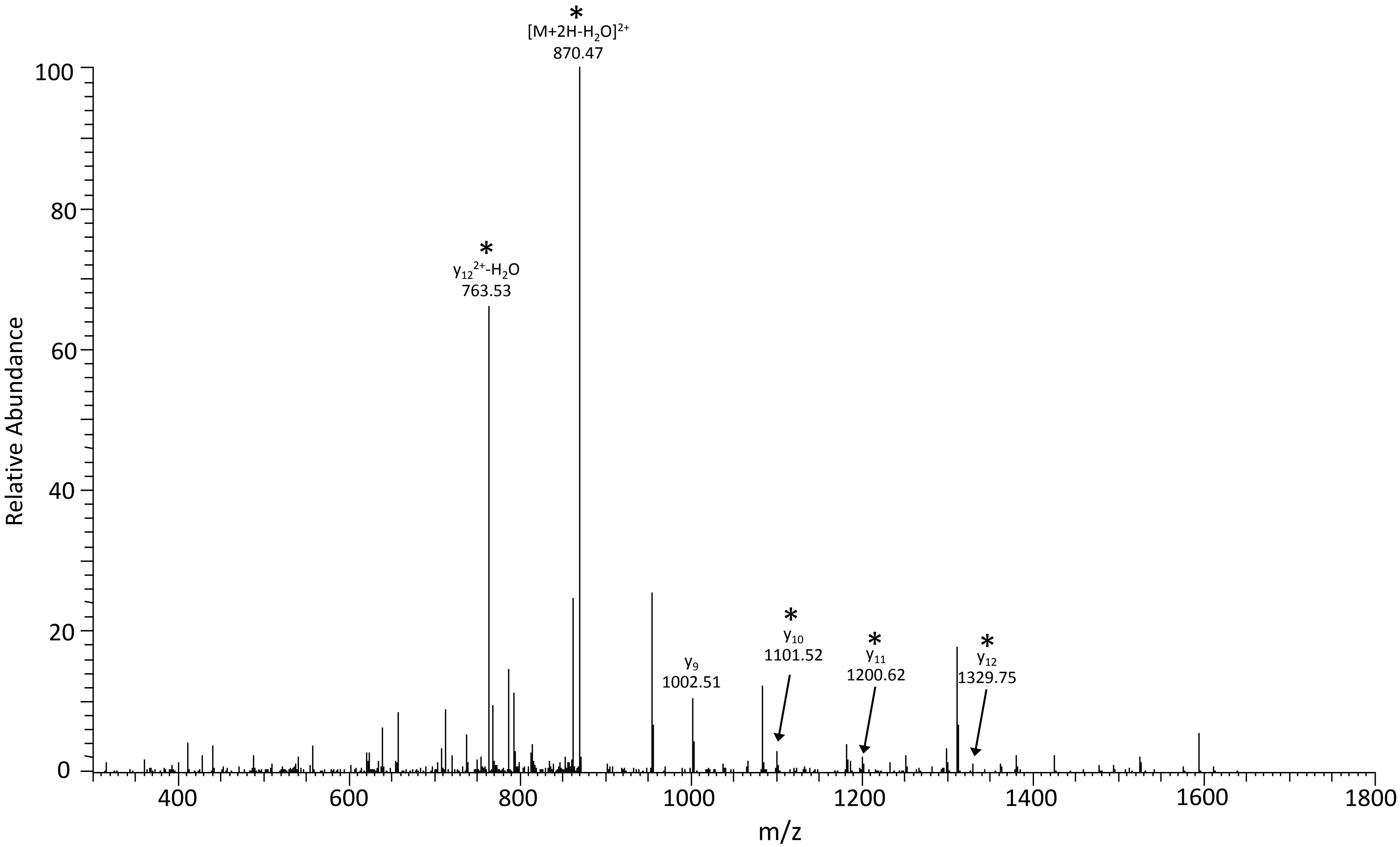

Sequence	Modifications	XCorr	Charge	m/z (Da)	MH+ (Da)	Δm (ppm)	t_r (min)	Enzyme
TLSDYNIQk	K^{63}-Oxidation	2.56	2	549.2772	1097.5472	-0.17	21.30	Trypsin

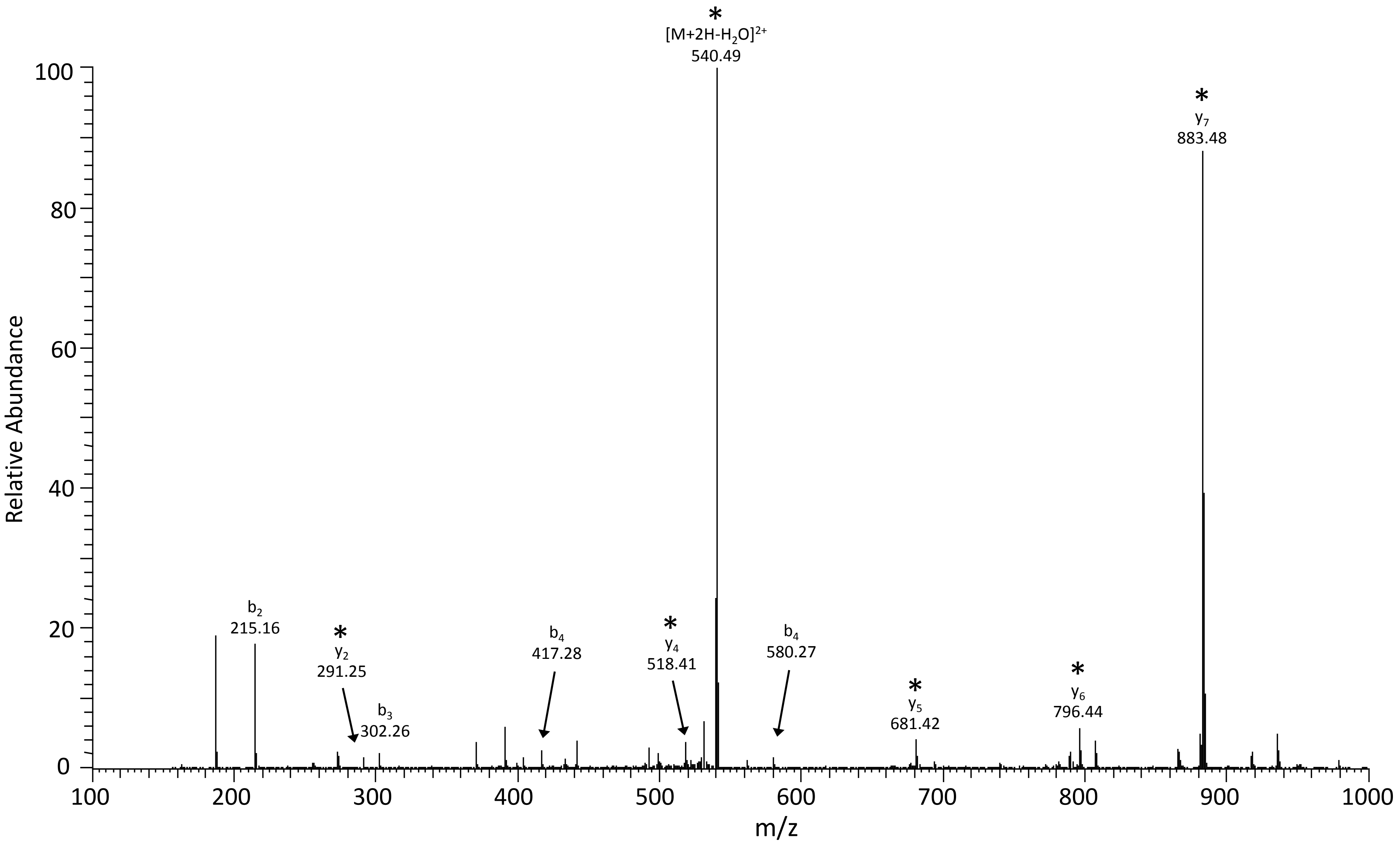

Sequence	Modifications	XCorr	Charge	m/z (Da)	MH+ (Da)	Δm (ppm)	t_r (min)	Enzyme
TLSDYNIqK	Q^{62}-Deamidation	2.17	2	541.7697	1082.5320	-4.09	14.47	Trypsin

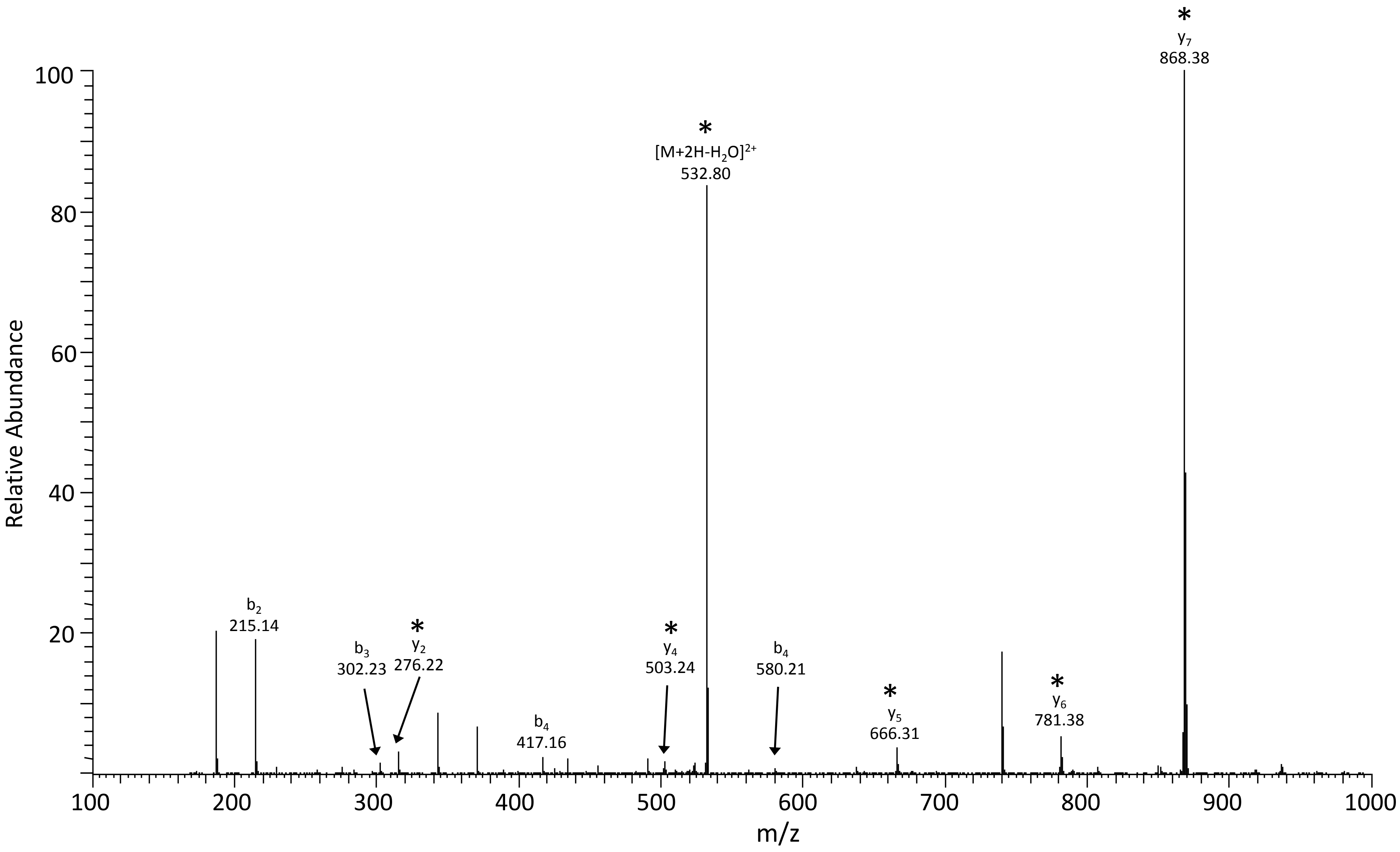

Sequence	Modifications	XCorr	Charge	m/z (Da)	MH+ (Da)	Δm (ppm)	t_r (min)	Enzyme
tLSDYNIQK	T^{55}-Oxd'n	2.13	1	1079.5325	1079.5325	-4.00	19.29	Trypsin

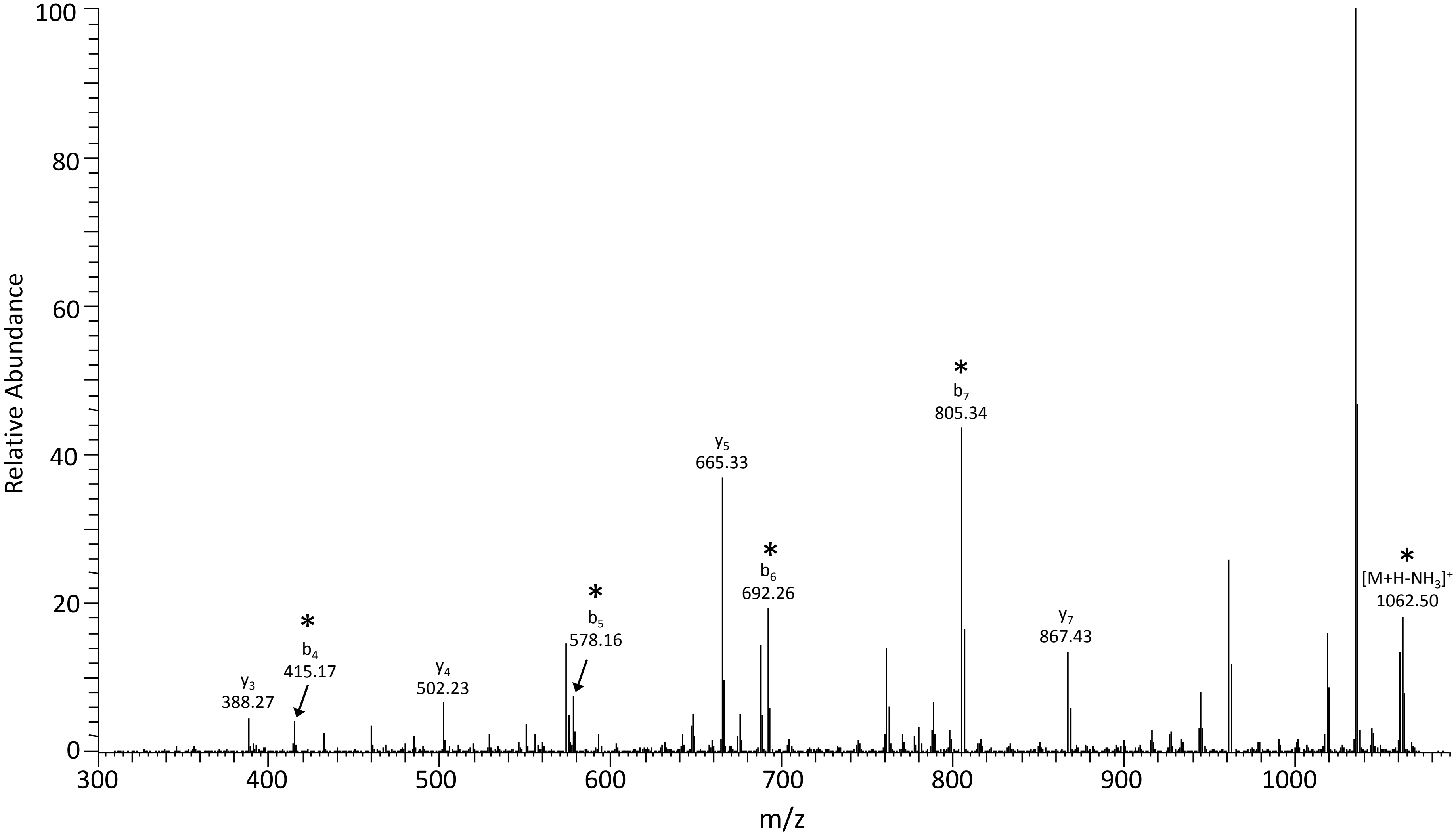

Supplement: S1 Fig — (PDF) [file pone.0116606.s001.pdf]
